# Genomic Analysis of Invasive Human Bone Marrow Derived Mesenchymal Stem Cells

**DOI:** 10.4172/2329-8820.1000122

**Published:** 2013-05-23

**Authors:** Lesley A Mathews, Elaine M Hurt, Xiaohu Zhang, William L Farrar

**Affiliations:** 1 Division of Preclinical Innovation, National Center for Advancing Translational Sciences, National Institutes of Health, Bethesda, MD 20892, USA; 2 Med Immune, LLC, Gaithersburg, MD 20878, USA; 3 Cancer Stem Cell Section, Laboratory of Cancer Prevention, Center for Cancer Research, Frederick National Laboratories, Frederick, MD 21702, USA

**Keywords:** hMSCs, Invasion, Gene expression, STAT3

## Abstract

**Background:**

Human bone marrow derived mesenchymal stem cells (hMSCs) are capable of differentiation into multiple cell lineages and demonstrate a wide variety of use in various therapeutic applications. Only recently has research begun to understand the gene expression profiles of hMSCs and their differentiated counterparts *in vivo* and *ex vivo*.

**Purpose:**

The research presented here aimed at gaining a better understanding of gene expression patterns present during hMSC invasion through a basement membrane.

**Methods:**

Changes in gene expression were evaluated between invasive and non-invasive cells using Agilent’s gene expression arrays and Matrigel invasion chambers. The cells were specifically attracted to a defined stem cell media called SCM.

**Results:**

A total 435 genes were up-regulated by 2- fold or more in the invasive population of cells and classified into developmental programs and immunological/inflammatory signaling pathways determined by Ingenuity Pathway Analysis (IPA). This list included a variety of regulators of growth and differentiation including NANOG, STAT3 and STAT5A and members of the polycomb repressive complex-2 (PCRC2) EZH2 and SUZ12. The known regulator of inflammation and hypoxia HIF-1α was also increased suggesting that regulation of the microenvironment is important during this process. Finally, the invasion process could be reversed using the STAT3 inhibitor Static.

**Conclusions:**

Overall these data will increase the understanding of the genetic pathways functioning during hMSC invasion and aid in the development of their therapeutic applications.

## Introduction

Adult human bone marrow derived mesenchymal stromal cells (hMSCs), also called mesenchymal stem cells, were first identified as highly proliferative/radiation resistant cells isolated from human bone marrow transplants [1]. These cells are capable of undergoing self-renewal and are multipotent, meaning that under the appropriate stimuli are capable of differentiating into multiple cell lineages including osteoblasts, chondrocytes, adipocytes and myoblasts [2–4]. With their diverse potential, these cells demonstrate a wide variety of use in many therapeutic applications such as osteogenesis imperfecta [5], myocardial infarction [6], muscular dystrophy [7], spinal cord injury [8,9], graft versus host disease [10,11] and have even recently been examined for their role in the treatment of cancer [12–14]. Previous research has characterized that injured or wounded tissues produce inflammatory cytokines which seem to attract hMSCs expressing CD29, CD44, CD51, CD73, CD105, CD166 and Stro-1 (bone morphogenic protein-3: BMP3) [15,16]. The expression of certain markers, however, does seem to be specific to the microenvironment. Additionally, the process of hypoxia, which is a reduction in the amount of available oxygen, is exhibited by a variety of tumors.

This process is regulated by hypoxia-inducible factor-1 α (HIF1α) [17] and injured tissues or tumors which are present in a hypoxic microenvironment produce these same angiogenic and inflammatory cytokines, further perpetuating the homing of hMSCs. Stem cell niches are often located in anatomical regions characterized by hypoxic conditions as they require low levels of oxygen to minimize damage caused by DNA oxidation. The relationship between oxygen and MSCs is under intense investigation as MSCs reside in locations close to the vascular structures, yet the tissues where MSCs are found exhibit low oxygen levels [18]. The exact mechanism(s) by which oxygen regulates MSCs is unknown, but it is clear that oxygen is a critical regulator of MSC fate.

The research presented here is aimed at gaining a better understanding of what these additional pathways regulating hMSCs might be. An in depth investigation of gene expression patterns present during hMSC invasion through a basement membrane was performed using a 44,000 probe-based gene expression array. These data have provided a unique gene expression signature of invasion toward a stem cell niche.

The research presented here is aimed at gaining a better understanding of what these additional pathways regulating hMSCs might be. An in depth investigation of gene expression patterns present during hMSC invasion through a basement membrane was performed using a 44,000 probe-based gene expression array. These data have provided a unique gene expression signature of invasion toward a stem cell niche.

## Methods

### Cell lines and reagents

Early passage (P3) human bone marrow derived mesenchymal stem cells (hMSCs) were obtained from Lonza (Gaithersburg, MD) and maintained using their recommended conditions with addition of 500 U/mL penicillin/500 μg/mL streptomycin and 0.250 μg/mL amphotericin-B all from Gibco (Invitrogen, Carlsbad, CA). The cultures were maintained in 5% CO2 air at 37°C. The STAT3 inhibitor Stattic (Calbiochem, Gibbstown, NJ) was dissolved in molecular grade ethanol.

### Matrigel invasion assay

Matrigel-coated 24-well inserts (8 μM pore size) and non-coated control inserts purchased from BD Biosciences Clontech (Palo Alto, CA) were used according to manufacturer’s instructions. Between 60,000 to 100,000 cells were seeded for the invasion in serum-free RPMI and migrated toward media specific for stem cells (SCM) containing DMEM/F12 with human supplementation of 10 ng/mL bFGF, 20 ng/mL EGF and 5 μg/mL insulin along with 0.4% BSA (each from Sigma, St. Louis, MO). Routine invasion assays were performed for 24 hours and then stained with the Diffi-Quick Staining kit (Dade Behring, Deerfield, IL). The direct STAT-3 inhibitor Static was used at 1 μM. Three to five microscopic fields (20X) were photographed and counted for each sample. The experiment was repeated three independent times.

### Microarray Analysis

RNA was isolated and labelled as previously described from invasive and non-invasive cells [19], with the following modifications: Reverse transcriptase was heat inactivated at 65°C for 10 minutes and again 100 ng of RNA was amplified using the Message Amp a RNA Amplification Kit. A total of 2 μg of Universal Reference RNA (Stratagene, La Jolla, CA) was labelled with Cy3-dUTP and experimental samples were labelled with Cy5-dUTP. Samples were hybridized to an Agilent whole genome gene expression array following manufacturer’s directions. Arrays were scanned using a GenePix 4000B scanner and extracted using the mAdb portal from the National Cancer Institute. The data was normalized using the Loess method and bad/or not found spots were excluded for extraction. The array data was further analyzed using Cluster and Tree view offered by Michael B. Eisen as freeware (http://rana.lbl.gov/EisenSoftware.htm) to produce the heat map. The complete list of genes whose expression changed ≥ 2- or ≤ 2- fold is available in Supplementary Table S1.

### Proliferation Assays

Cells were seeded overnight in a 96 well plate in 100 μL of regular media at a density of 2000 cells per well. Cells were treated with either DSMO or 1 μM Stattic. Cell proliferation was measured using the CellTiter-Glo assay from Promega (Madison, WI, USA) after 24 hours using 100 μL of reagent and an incubation time of 20 minutes. The relative luciferase units (RLU) were quantified using a Tecan Infinite 200 plate reader. Samples were performed using an N of 8.

### Quantitative Real Time Polymerase Chain Reaction (QRT-PCR)

Total RNA was isolated using TRIzol (Invitrogen Corporation, Carlsbad, CA). RNA from ‘top’ cells was isolated using a cell pellet acquired from trypsinizing cells from one membrane after bottom cell were removed with a cotton swab. Conversely, RNA from the bottom cells was isolated by combining three membranes where the top cells were removed using a cotton swab. The membranes were pooled and placed in TRIzol for 10 minutes at room termperature, and the conventional procedure for isolation of RNA was then followed. To increase the yield of RNA, 5 μg of linear arcylamide (Ambion, Austin, TX) was added prior to precipitation of RNA with isopropanol. Additional to increase overall yield, 100 ng of RNA was amplified using the Message Amp aRNA Amplification Kit (Ambion, Austin, TX). cDNA was prepared using the SuperScript®III First-Strand Synthesis System (Invitrogen Corporation, Carlsbad, CA). Quantitative real time polymerase chain reaction (QRT-PCR) analysis was performed using a StepOne Real-time PCR machine (Applied Biosystems, Foster City, CA) with TaqMan Gene Expression Assay reagents and probes (Applied Biosystems). A total of 4 μL of cDNA was used in a 20 μL reaction resulting in a 1:5 dilution. The following SYBR based human probes were used: ER-β (Hs01112040_m1), EZH2 (Hs00544830_ m1), HIF1α (Hs00153153_m1), NANOG (Hs02387400_g1), STAT3 (Hs01047580_m1), STAT5A (Hs00559643_m1), SUZ12 (Hs00248742_ m1) and 18S rRNA (Hs99999901_s1). Relative fold induction of mRNA was compared between top and bottom cells using the Delta-Delta CT method of quantitation, and 18S rRNA was used as a loading control.

### Ingenuity and Genomatrix Software

Agilent databases (excel 2003 format) were uploaded for analysis using software available at http://www.ingenuity.com/ or http://www.genomatix.de/

### Statistical Analysis

Using Graph Pad Prisim (Version 4) a Two-way ANOVA with a Bonferroni post test was performed to compare groups and * represents a p-value of <0.05 comparing invasive to non-invasive cells.

## Results

### Microarray analysis of invasive hMSCs

**Figure 1:**
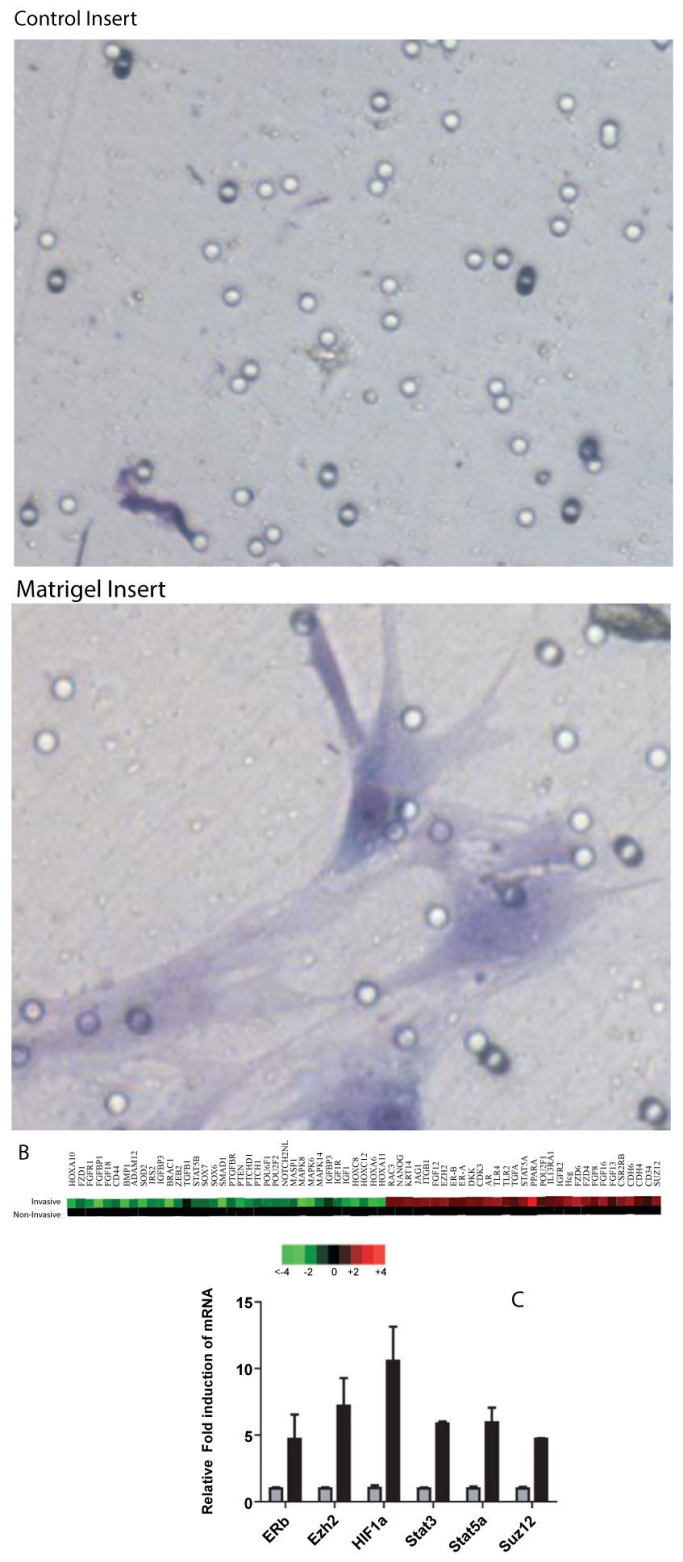
Gene expression changes in invasive hMSCs **A)** Microscopic images (20X) of cells invaded toward SCM after 24 hours using either control inserts or inserts containing Matrigel. Cells were staining with the Diffi-Quick Staining kit. **B)** Heat Maps demonstrating increased (red) or decreased (green) expression of a select number genes from the invasive compared to non-invasive cells. Samples were hybridized to an Agilent whole genome gene expression array following manufacturer’s directions. Arrays were scanned using a GenePix 4000B scanner (Molecular Devices, Sunnyvale, CA) and analyzed using Cluster and Treeview offered by Michael B. Eisen as freeware (http://rana.lbl.gov/EisenSoftware.htm). The complete list of genes whose expression changed ≥1.8- or ≤1.8-fold is available in Supplemental Table S1. **C)**A select number of up-regulated genes were verified using qRT-PCR for increased expression in the invasive cells. Gray bars represent the non-invasive cell expression normalized to 1 and black bars are relative fold-induction of mRNA from invasive cells. Fold induction was calculated using the Delta-Delta CT method where the non-invasive cells were set at 1.0 as the control, and 18S rRNA was used as a loading control. Data is shown as transformed log2. A Twoway ANOVA with a Bonferroni post-test was performed to compare groups and * represents a p-value of < 0.05 comparing invasive to non-invasive cells.

Initially it was determined that a small percentage (~20%) of hMSC were able to invade though the Matrigel toward a highly defined media called stem cell media (SCM) previously shown to attract invasive prostate cancer stem cells [19–21] (Figure 1A). A very small number of cells were able to migrate across the control membrane toward SCM, demonstrating the importance of Matrigel (or a basement membrane) in hMSC homing. To determine which genes are differentially regulated within this population of invaded cells compared to the non-invasive cells, a gene expression microarray was performed using Agilent whole genome expression array (1×44K). Arrays were extracted using the mAdb Gateway from the National Cancer Institute (NCI) and after background subtraction and normalization to universal RNA, a total of 435 genes were found to be up-regulated by 2-fold or more in the invasive population of cells (Supplemental Table 1).

Heat maps generated by using TreeView demonstrated the variety of genes related to ‘stemness’ and migration/invasion/metastasis (Figure 1B and 1C) which increased in the invasive cells. When carefully analyzing the specific genes in the array that are increased after invasion (Table 1), a variety of receptors were found to up-regulated including androgen receptor (AR), estrogen receptor-β (ER-β or ESR2) and a number of immune based receptors increased including interleukin-13 receptor-α1 (IL13RA1) and toll-like receptor-2 and 4 (TLR2 and TRL4). Growth and differentiation genes such as fibroblast growth factor-12 (FGF12) and insulin-like growth factor-2 receptor (IGF2) were also increased. The stem cell regulator NANOG and additional genes implicated in regulation of stem cells including cytokeratin-14 (KRT14), intergrin- β1 (ITGβ1), the Ras protein ras-related C3 botulinum toxin substrate-3 (RAC3), the notch-1 receptor ligand jagged-1 (JAG1) and finally the signal transducer and activator of transcription-5A (STAT5A) were also increased. Two members of the polycomb repressive complex-2 (PCRC2) enhancer of zeste-2 (EZH2) and suppressor of zeste-12 (SUZ12) were increased as well.

### qRT-PCR confirmation of microarray data

Small panels of these genes were analyzed for confirmation of their expression patterns using qRT-PCR (Fig. 1C). The genes analyzed included a 4.7-fold increase in ER-β, as well as a 7.2- fold increase in the PCRC2 members EZH2 and 4.7-fold increase in SUZ12. Finally, the growth regulators STAT3 and STAT5A demonstrated a 5.8-fold and 5.9- fold increase, respectively, and although STAT3 was only up-regulated by 1.6-fold in the array we further analyzed the gene due its growing importance in stem cell maintenance. The stem cell gene NANOG was found to increased 2.29-fold in the array (Table 1), yet only 1.4-fold by qRT-PCR (data not shown).

### Ingenuity pathway analysis

**Figure 2:**
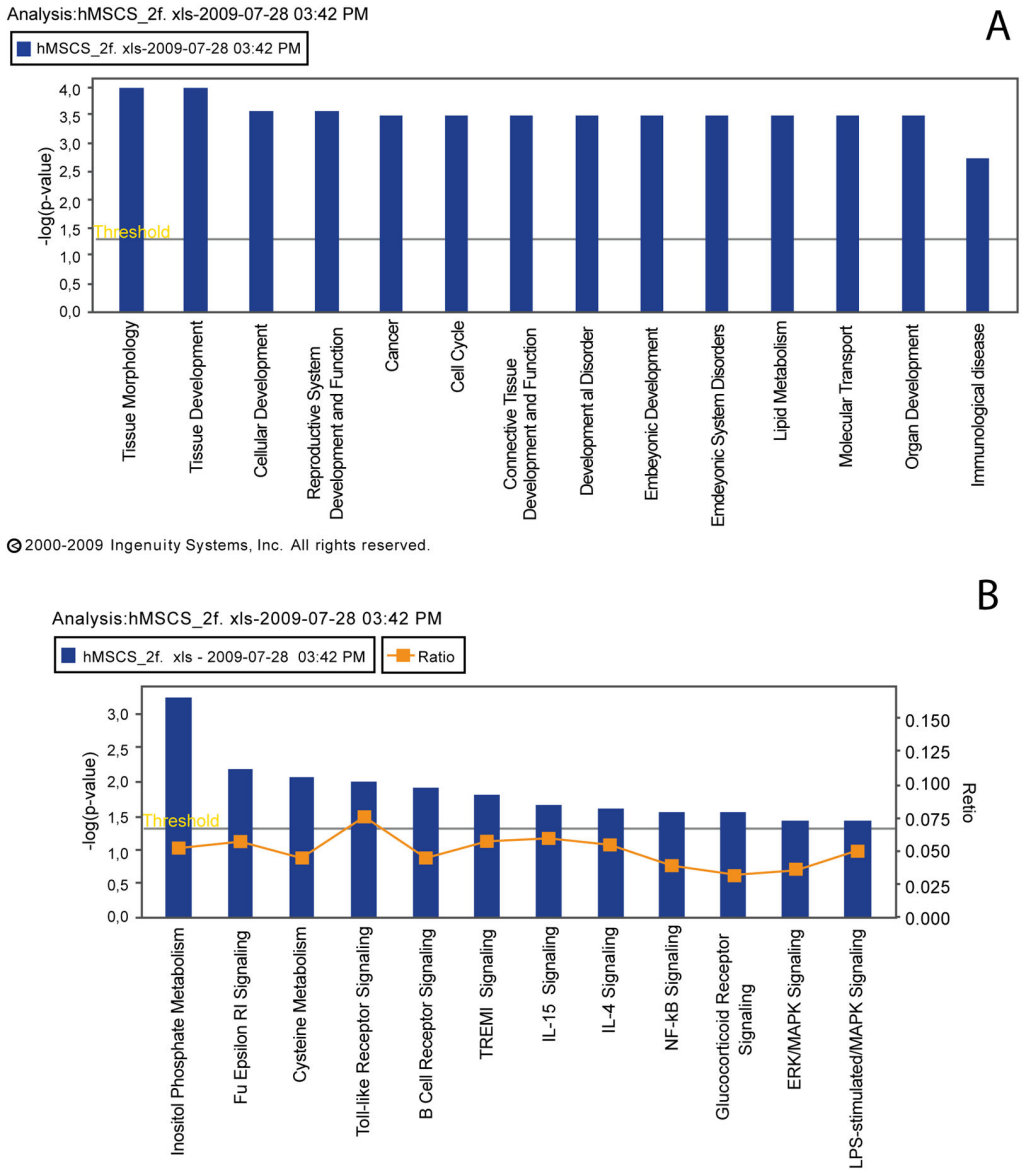
Ingenuity pathway analysis demonstrating significant changes (A) genes in developmental pathways (B) immunological based pathways in invasive hMSC cells. Any pathway above the yellow threshold bar demonstrates significant changes in gene expression

The majority of genes demonstrating an increase in expression classified into developmental programs including tissue development, cellular development, reproductive system development, connective tissue development, embryonic development and organ development (Figure 2A). A number of genes also belong to the immunological disease category (Figure 2A) and the canonical immunological/inflammatory signalling pathways including Fc Epsilon RI signalling, Toll-like receptor signalling, B-Cell receptor signalling, TREM1 signalling, IL-15 and IL-4 signalling and finally the NFκB pathway (Figure 2B).

### STAT3 regulates hMSC invasion

**Figure 3:**
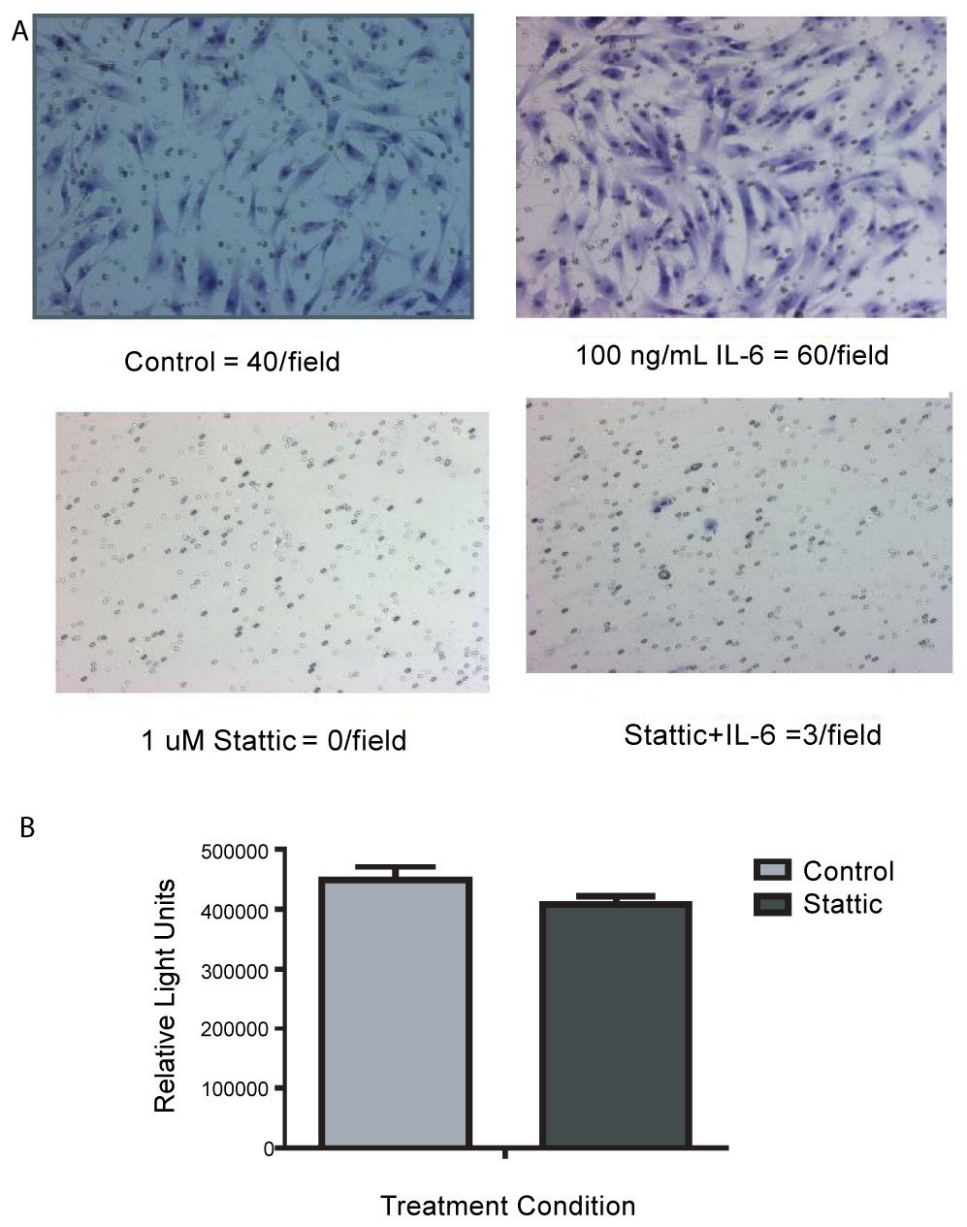
Inhibition of invasive hMSCs toward SCM using the STAT3 inhibitor Stattic **A)** Matrigel invasion assays were seeded with 60,000 cells per well in RPMI and invaded toward SCM with or without 100 ng/mL of IL-6, and with or without 1 μM Stattic. After 24 hours the cells were stained using the Diffi-Quick Staining kit and 4 independent fields were counted and averaged. The experiment was repeated twice and each time no cells were able to invade toward SCM in the presence of Stattic. **B)** Cell-titer glow experiment for cell proliferation of hMSCs treated with or without 1 μM Stattic for 24 hours

Recent data suggests a number of these pathways that are increasing with invasion of hMSCs intersect using the STAT3 pathway [22–25]. Using this same invasion assay, our lab has recently demonstrated that the invasion of prostate cancer cells toward SCM can be blocked using the STAT3 inhibitor Stattic [26]. This prompted us to test if Stattic could also block invasion of hMSCs toward SCM. In the presence of Stattic, invasion of the hMSCs was completely abolished without affecting normal cell proliferation, suggesting a strong role for the STAT proteins in mediating this process (Figure 3A and 3B).

## Discussion

Human bone marrow derived mesenchymal stem cells (hMSCs) have the potential to revolutionize medicine with their donor specific treatment of diseases. hMSCs represent an attractive model for a number of reasons, including the lack of ethical controversy regarding their isolation since the cells themselves can be isolated from the patient’s own blood/tissues [1], as well as their lack of significant immunogenicity allowing for allogenic transplantation without the use of potentially harmful immunosuppressive drugs [27]. Albeit as provocative as this system may seem there is still much unknown about the behaviour of these patient derived hMSCs. In order to begin to determine which genes might be differentially regulated during the process of hMSCs invasion we analyzed changes in gene expression patterns of the cells invading through a basement membrane of Matrigel. Using the defined media SCM we were able to identity a specific population of invasive hMSCs moving across the Matrigel membrane. This demonstrates that only a portion of the MSCs have the ability to be attracted by the SCM. Clearly, it can be assumed that the invasive cells express receptors for the components of the SCM, yet there is a significant amount of cross-talk and activation that occurs in response to signalling by each EGF, bFGF and insulin independently and synergistically when used in combination. From the microarray, we found that 435 genes were up-regulated by 2-fold or more after normalization and background subtraction. An increase in a number of classical chemokine receptors such as IL13RA1and TLR2 and TLR4 was observed, in addition to an increase in AR and ER-β. Traditionally these sex hormone receptors were not thought to be expressed on stem cells, but in a recent review by Ray et al., the authors summarize their roles in the differentiation of hMSCs and embryonic stem (ES) cells [28]. For example, in four independent mouse ES cell line there is functional expression of AR which can interact with transfected androgen response elements [29]. In addition, treatment of hMSCs with 17β-estradiaol is able to help induce osteogenic differentiation [30]. Furthermore, AR expression has been validated on CD34+ cells [31] and with regards to more tissue specific stem cells, AR expression has been seen in putative stem cell population from prostate tumor lines [32]. The increased expression of these sex steroid hormone receptors within the invasive population of hMSCs could be utilized for further differentiation of the cells depending on the environment and/or niche to which they are homed to. The increased expression of TLRs are very interesting in the context of this study. Toll-like receptors (TLRs) involved in mediating stress responses of hMSCs occur through the activation of many pathways, including NFκB, AKT and MAPK [33]. Additionally, activation of TLRs with their various ligands, including lipopolysaccharide (LPS)-induced expression of a variety of chemokines and cytokines including C-X-C motif chemokine 10 (CXCL10), interferon-1β (INF1β) and interleukin-6 (IL-6) resulted in an enhanced migratory ability [33]. Furthermore, the importance and role of TLRs, as well as other growth hormones, chemokine/cytokines receptors and adhesion molecules expressed by hMSCs is summarized nicely by Spaeth et al. [16] in a review regarding ‘the migratory itinerary of mesenchymal stem cells.’ Observing an increased expression of TLR2 and TLR4 in the invasive cells was not surprising based on this evidence. Evolutionarily conserved co-expression of transcription factors in human ES cells have been investigated for a number of years, and of interest to our research is the overlap in expression of the such genes as NANOG and STATs [34]. Our data demonstrates an increase in each of these gene families within the invasive population of hMSCs. As previously mentioned, we observed a significant increase in STAT5A within the invasive cells, and a slight increase in STAT3, both of which were validated by qRT-PCR. The role of STATs in the regulation of stem cells has been investigated for a number of years, but only recently has their role in the regulation of hMSCs, tissue specific stem cells and invasion surfaced within the literature [24,25,35–41]. To further determine the importance of STATs/IL-6 since this has been a longstanding interest in our lab, we placed the STAT3 specific inhibitor Stattic in the chemoattractive media and we observed a complete block in invasion (Figure 3). Although addition of exogenous IL-6 did slightly increase invasion, the presence of this inhibitor was again able to block this invasion (Figure 3). The observation that inflammatory regulator HIF-1α was also increased in the invasive cells was of interest and in direct relation to the STAT data. Recently, it has been shown that HIF-1α regulates IL-6 in low oxygen environments, and IL- 6 increases when HIF-1α is active [42]. A recent review relates the relationships of HIFs with such pathways governing stem cell regulation such as octamer binding transcription factor 4 (OCT4), bone morphogenic proteins (BMPs), NOTCH, sonic hedgehog (SHH) and β-catenin/WNT [43]. Thus, taken together with our data, we believe that IL-6/STAT plays a key role in hMSC invasion and homing and should be taken into consideration when developing therapeutic uses of hMSCs. Additionally, selective withdrawal of IL-6 and induction with tissue specific factors could ensure proper timing and specificity of hMSCs differentiation. Additional changes we observed that we believe are important in the regulation of hMSC homing and invasion based on previous research by our lab and others include increases in gene members of the Glinsky Signature (SUZ12 and EZH2), which determine likelihood of death from metastatic disease [44–46]. EZH2 is involved in histone methylation and deacetylation in stem cells and a recent paper shows that retroviral overexpression of EZH2 in mouse embryonic fibroblasts (MEFs) resulted in bypassing of the senescence program [47]. With regards to normal HSCs, which were rapidly exhausted after serial transplantations, overexpression of EZH2 completely conserved long-term repopulating potential [47]. Finally, recent data suggests that EZH2 regulated by STAT3 is correlated to the pathological stage and progression of prostate cancer [48].

## Conclusion

Overall, we have determined that a number of genes involved in immunological maintenance, developmental processes and the regulation of ‘stemness’ are increased in invasive hMSCs. By understanding the pathways functioning during hMSC invasion we believe this will aid in the development of their therapeutic applications.

## Supplementary Material

Supplementary file

## Figures and Tables

**Table 1:** Genes up-regulated in invasive hMSC cells determined by microarray analysis Significant changes in selected genes demonstrating a 1.6-fold or higher increase. The Agilent ID corresponds to the probe ID from the array, values for non-invasive and invasive hMSCs as well as their fold change (Invasive-Non-Invasive) are provided.

Agilent ID	hMSC Non- Invasive	hMSC_Invasive	Fold Change	UNIQID	Gene Name
A_24_P919916	−1.7055	5.8604	7.5659	WID:6488014	PTF1A
A_23_P42065	−2.8123	0.9734	3.7857	WID:6484664	TNFRSF21
A_23_P30736	−2.4033	0.8032	3.2065	WID:6496619	HLA-DOB
A_23_P60306	−1.43	1.7307	3.1607	WID:6502707	TLR4
A_23_P113111	−1.7102	1.2511	2.9613	WID:6488464	AR
A_23_P77223	−1.0517	1.9013	2.953	WID:6513234	MESP1
A_23_P210763	−2.7955	0.1168	2.9123	WID:6486716	JAG1
A_24_P38387	−3.0463	−0.1468	2.8995	WID:6504089	NDRG1
A_32_P122579	0.6126	3.5082	2.8956	WID:6480399	EZH2
A_23_P207367	−2.2092	0.5955	2.8047	WID:6481655	STAT5A
A_23_P84320	−1.8188	0.8944	2.7132	WID:6495148	HMX1
A_24_P265346	−2.8338	−0.1382	2.6956	WID:6504873	KRT14
A_23_P77440	−2.7784	−0.1771	2.6013	WID:6502102	NFATC3
A_32_P160537	2.0805	4.6637	2.5832	WID:6492904	FGF12
A_23_P206441	−2.1401	0.3806	2.5207	WID:6586963	FANCA
A_24_P598836	1.5009	3.9523	2.4514	WID:6517296	ITGB1
A_23_P214011	−1.9233	0.4855	2.4088	WID:6494488	CDH6
A_23_P23947	−1.2674	1.096	2.3634	WID:6489062	MAP3K8
A_23_P156953	−1.7175	0.6025	2.32	WID:6517216	IGF2R
A_23_P125001	−0.4863	1.8332	2.3195	WID:6482994	RAC3
A_23_P204640	−4.0766	−1.7784	2.2982	WID:6487751	NANOG
A_23_P92499	0.3371	2.5755	2.2384	WID:6486692	TLR2
A_24_P889103	−2.3142	0.6211	2.9353	WID:6516766	SUZ12P
A-23_P54100	−1.5936	0.4465	2.0401	WID:6506930	ESR2
A_23_P137196	−1.3207	0.6947	2.0154	WID:6488748	IL13RA1
A_23_P46964	0.0039	1.6847	1.6808	WID:6478939	HIF1AN
A_23_P100795	−0.1833	1.42	1.6083	WID:6480615	STAT3

## Abbreviations

hMSCs: Human Bone Marrow Derived Mesenchymal Stem Cells
SCM: Stem Cell Media
qRT-PCR: Quantitative Real Time Polymerase Chain Reaction
IPA: Ingenuity Pathway Analysis
STAT3: Signal Transducer And Activator Of Transcription 3
STAT5A: Signal Transducer And Activator Of Transcription 5A
PCRC2: Polycomb Repressive Complex-2
EZH2: Enhancer of Zeste-2
SUZ12: Suppressor of Zeste-12
HIF-1α: Hypoxia- Inducible Factor-1 Alpha
EGF: Epidermal Growth Factor
FGF: Fibroblast Growth Factor
ERβ: Estrogen Receptor Beta
NCI: National Cancer Institute
AR: Androgen Receptor
IL13RA1: Interleukin-13 Receptor Alpha-1
TLR2/4: Toll-Like Receptor-2/4
FGF12/7: Fibroblast Growth Factor-12/716
IGF2: Insulin-Like Growth Factor-2
KRT14: Cytokeratin-14
ITGβ1/4: Intergrin Beta-1/4
RAC3: Ras Related C3 Botulinum Toxin Substrate-3
JAG1: Jagged-1
TREM1: Triggering Receptor Expressed On Myeloid Cells-1
IL: Interleukin
NFκB: Nuclear Factor Kappa B
ES: Embryonic Stem
AKT: Protein Kinase B
MAPK: Mitogen Activated Protein Kinase
INF1β: Interferon 1 Beta
CXCR4/10: C-X-C Chemokine Receptor Type-4/10
OCT4: Octamer-Binding Transcription Factor 4
BMPs: Bone Morphogenic Proteins
SHH: Sonic Hedgehog
WNT: Wingless-Type MMTV Integration Site Family
EPCs: Endothelial Precursor Cells
VCAM-1: Vascular Cellular Adhesion Molecule-1
MEF: Mouse Embryonic Fibroblast
