# Supplementary material for: Genomic Analysis of Invasive Human Bone Marrow Derived Mesenchymal Stem Cells
Source: J Bone Marrow Res. Author manuscript; Available in PMC 2014 Apr 25. (PMC3999892; doi:10.4172/2329-8820.1000122)
Supplement: Supplementary file [file NIHMS539034-supplement-Supplementary_file.pdf]

Supplementary Material

Additional File 1: xls

Supplementary Table S1: Total microarray gene list of up-regulated targets in invasive cells.

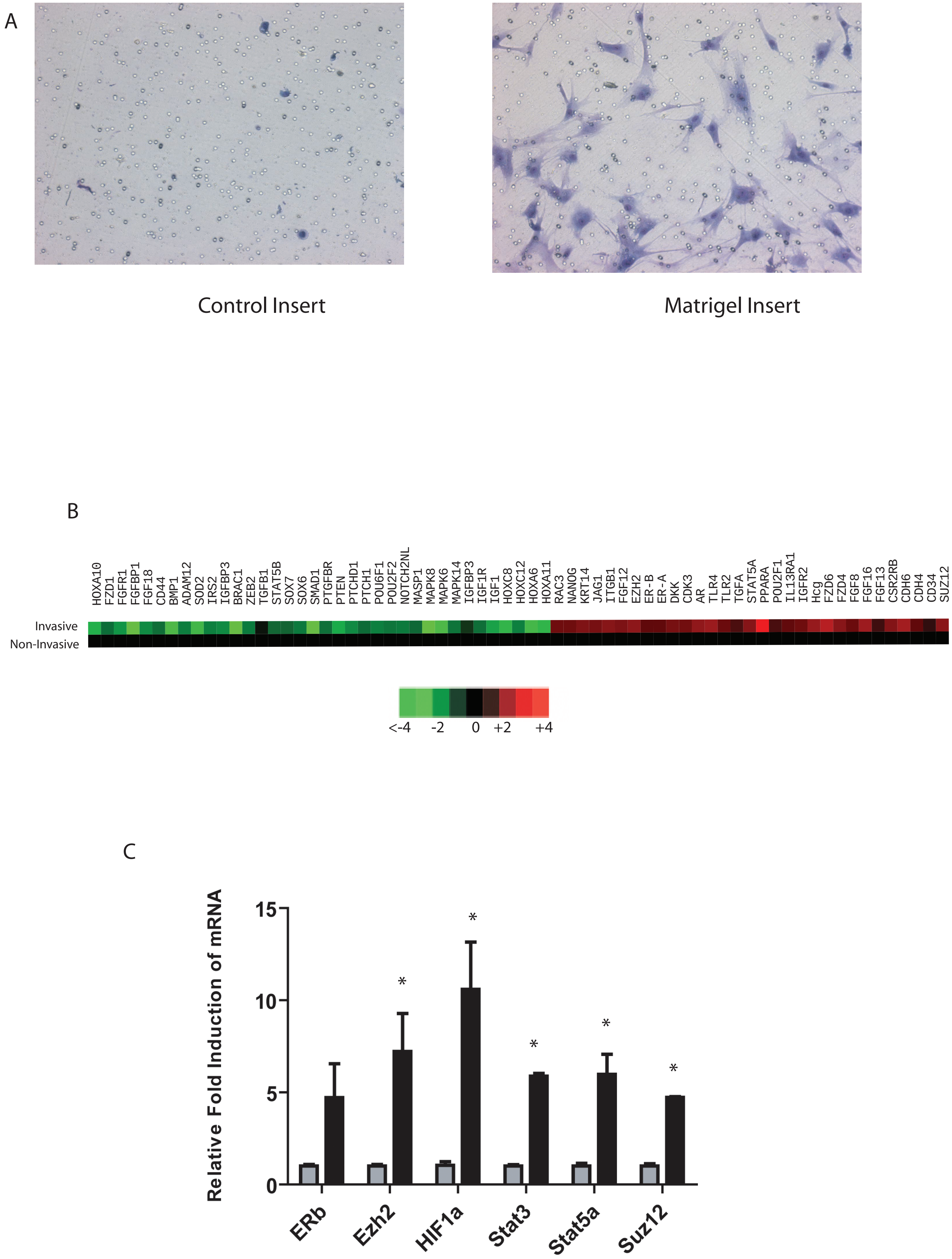
A
Control Insert
Matrigel Insert
B
HOXA10
FZD1
FGFR1
FGFBP1
FGF18
CD44
BMP1
ADAM12
SOD2
IRS2
IGFBP3
BRAC1
ZEB2
TGFB1
STAT5B
SOX7
SOX6
SMAD1
PTGFBR
PTEN
PTCHD1
PTCH1
POU6F1
POU2F2
NOTCH2NL
MASP1
MAPK8
MAPK6
MAPK14
IGFBP3
IGF1R
IGF1
HOXC8
HOXC12
HOXA6
HOXA11
RAC3
NANOG
KRT14
JAG1
ITGB1
FGF12
EZH2
ER-B
ER-A
DKK
CDK3
AR
TLR4
TLR2
TGFA
STAT5A
PPARA
POU2F1
IL13RA1
IGFR2
HCg
FZD6
FZD4
FGF8
FGF16
FGF13
CSR2RB
CDH6
CDH4
CD34
SUZ12
Invasive
Non-Invasive
<-4
-2
0
+2
+4
C
Relative Fold Induction of mRNA
15
10
5
0
*
*
*
*
*
ERb
Ezh2
HIF1a
Stat3
Stat5a
Suz12

A

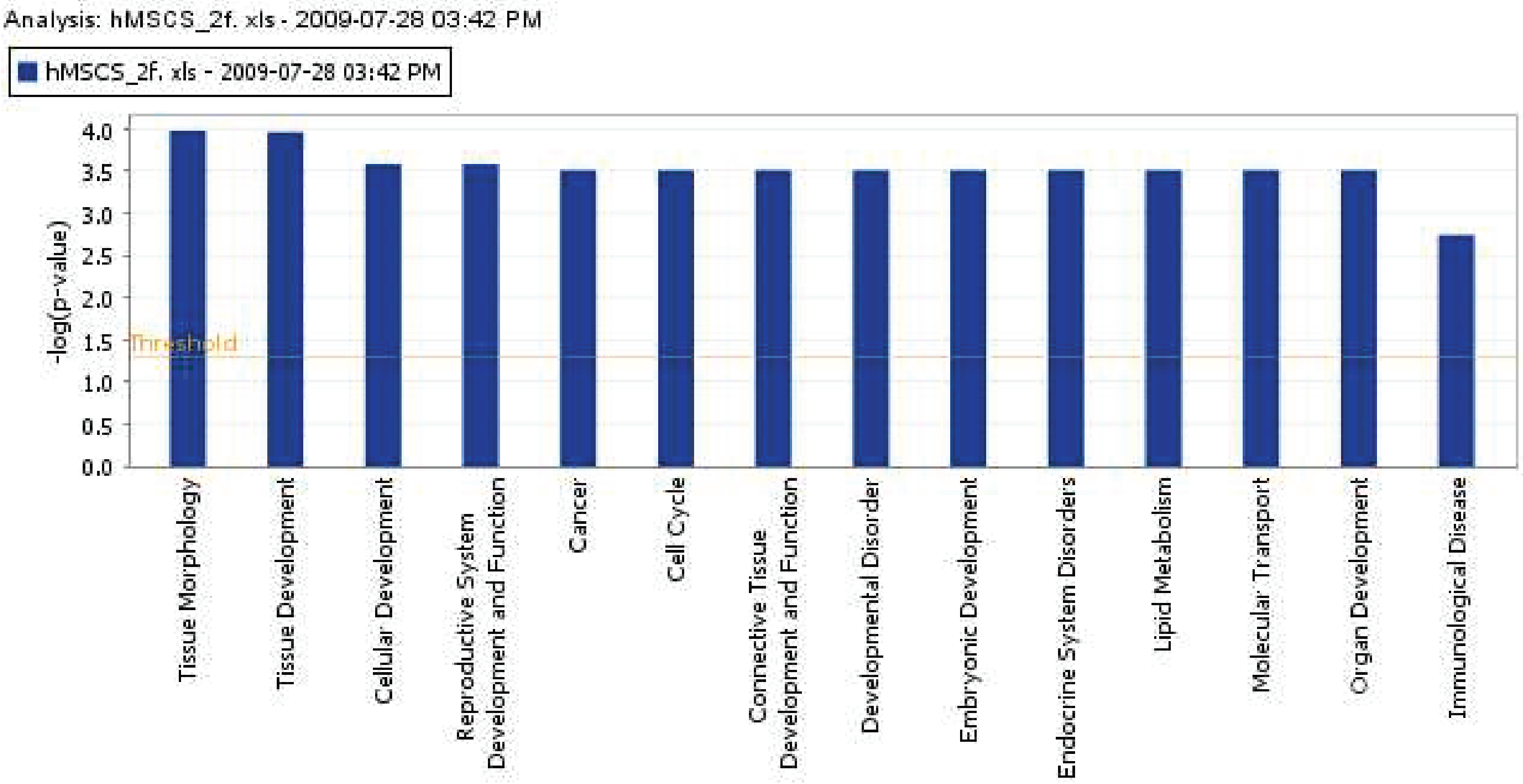

© 2000-2009 Ingenuity Systems, Inc. All rights reserved.

B

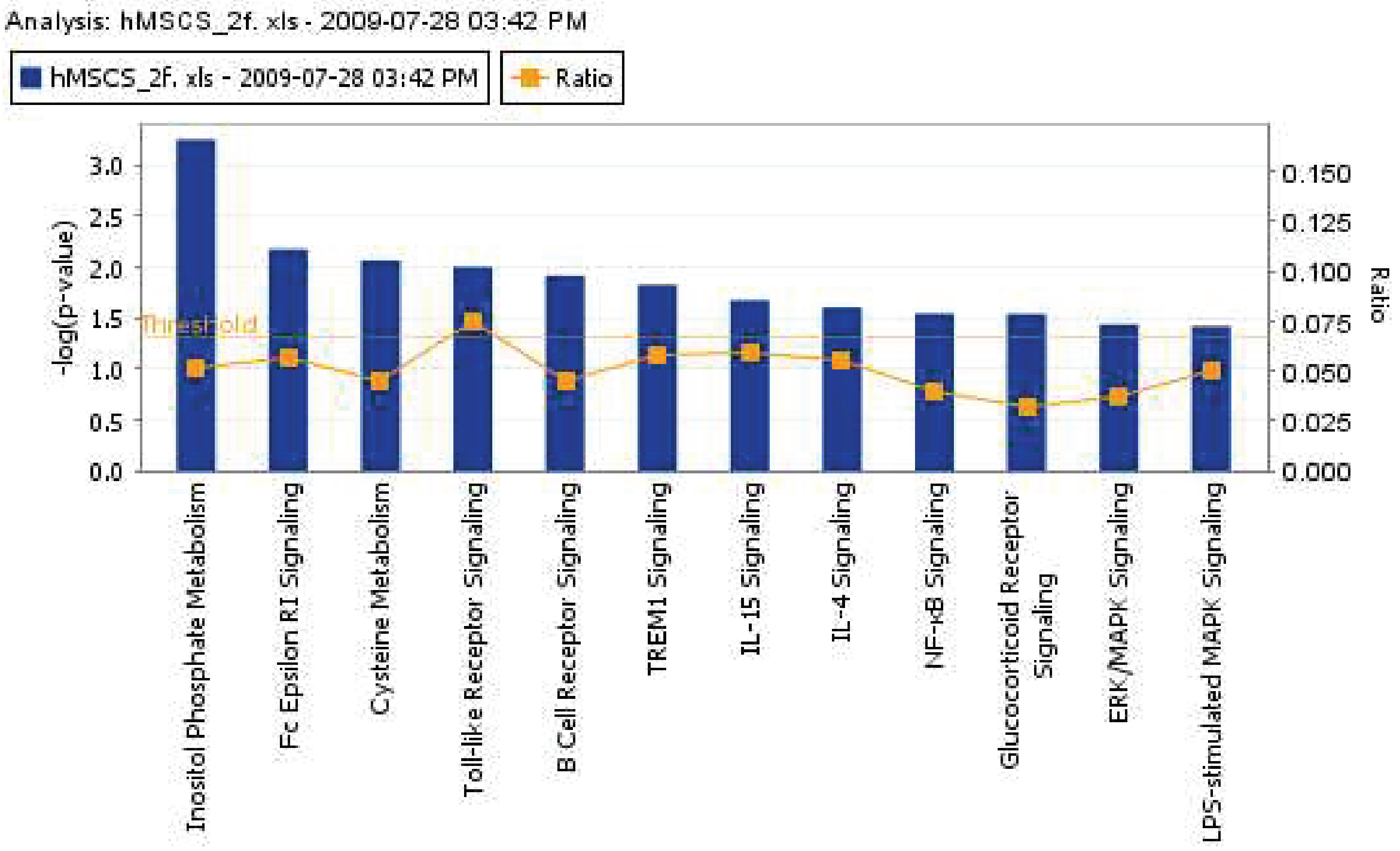

© 2000-2009 Ingenuity Systems, Inc. All rights reserved.

A

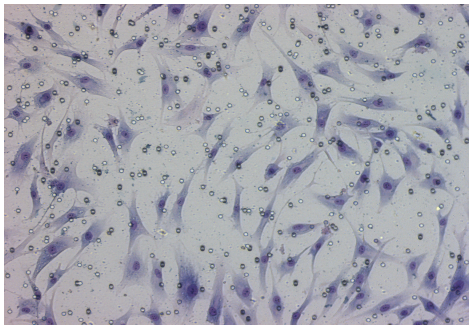

Control = 40/field

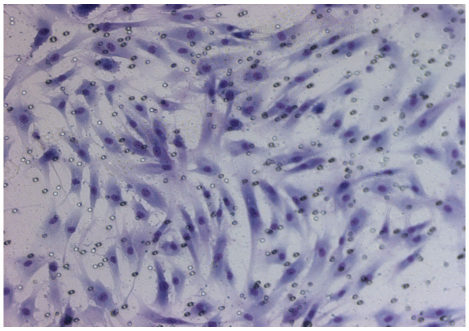

100 ng/mL IL-6 = 60/field

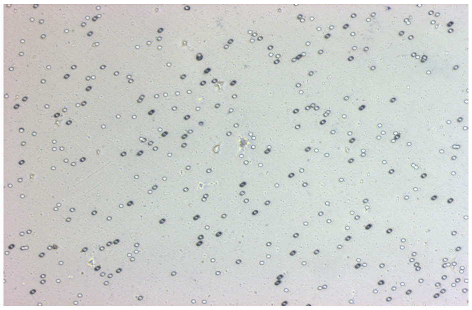

1 uM Stattic = 0/field

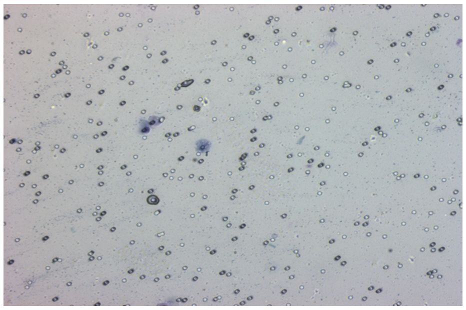

Stattic+IL-6 = 3/field

B

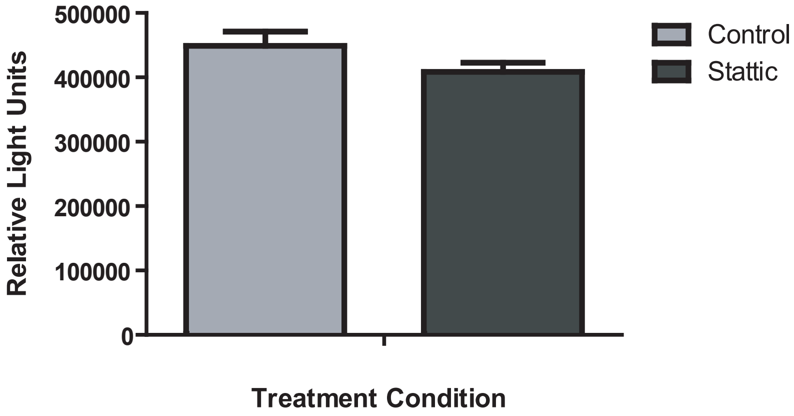

Table 1: Genes significantly up-regulated in invasive hMSCs.

Agilent ID	hMSC Non-Invasive	hMSC_Invasive	Fold Change	UNIQID	Gene Name
A_24_P919916	-1.7055	5.8604	7.5659	WID:6488014	PTF1A
A_23_P42065	-2.8123	0.9734	3.7857	WID:6484664	TNFRSF21
A_23_P30736	-2.4033	0.8032	3.2065	WID:6496619	HLA-DOB
A_23_P60306	-1.43	1.7307	3.1607	WID:6502707	TLR4
A_23_P113111	-1.7102	1.2511	2.9613	WID:6488464	AR
A_23_P77223	-1.0517	1.9013	2.953	WID:6513234	MESP1
A_23_P210763	-2.7955	0.1168	2.9123	WID:6486716	JAG1
A_24_P38387	-3.0463	-0.1468	2.8995	WID:6504089	NDRG1
A_32_P122579	0.6126	3.5082	2.8956	WID:6480399	EZH2
A_23_P207367	-2.2092	0.5955	2.8047	WID:6481655	STAT5A
A_23_P84320	-1.8188	0.8944	2.7132	WID:6495148	HMX1
A_24_P265346	-2.8338	-0.1382	2.6956	WID:6504873	KRT14
A_23_P77440	-2.7784	-0.1771	2.6013	WID:6502102	NFATC3
A_32_P160537	2.0805	4.6637	2.5832	WID:6492904	FGF12
A_23_P206441	-2.1401	0.3806	2.5207	WID:6586963	FANCA
A_24_P598836	1.5009	3.9523	2.4514	WID:6517296	ITGB1
A_23_P214011	-1.9233	0.4855	2.4088	WID:6494488	CDH6
A_23_P23947	-1.2674	1.096	2.3634	WID:6489062	MAP3K8
A_23_P156953	-1.7175	0.6025	2.32	WID:6517216	IGF2R
A_23_P125001	-0.4863	1.8332	2.3195	WID:6482994	RAC3
A_23_P204640	-4.0766	-1.7784	2.2982	WID:6487751	NANOG
A_23_P92499	0.3371	2.5755	2.2384	WID:6486692	TLR2
A_24_P889103	-2.3142	0.6211	2.9353	WID:6516766	SUZ12P
A-23_P54100	-1.5936	0.4465	2.0401	WID:6506930	ESR2
A_23_P137196	-1.3207	0.6947	2.0154	WID:6488748	IL13RA1
A_23_P46964	0.0039	1.6847	1.6808	WID:6478939	HIF1AN
A_23_P100795	-0.1833	1.42	1.6083	WID:6480615	STAT3

Agilent ID	hMSC Non-Invasive	hMSC_Invasive	Fold Change
A_24_P100368	-2.8141	2.5793	-0.2348
A_23_P375147	-1.3907	2.0936	0.7029
A_23_P40952	-0.3712	2.4003	2.0291
A_32_P201564	-1.5386	3.5079	1.9693
A_23_P348257	-3.6084	3.8299	0.2215
A_24_P938352	-0.9709	2.2552	1.2843
A_24_P917123	-2.8834	2.8254	-0.058
A_23_P153070	-2.5719	2.3637	-0.2082
A_24_P900730	-0.8469	2.2687	1.4218
A_23_P70328	-2.6156	2.1372	-0.4784
A_23_P6335	-2.7309	2.5134	-0.2175
A_24_P118489	-2.3329	2.3421	0.0092
A_24_P124325	-2.126	2.3518	0.2258
A_32_P6233	-2.3891	3.1514	0.7623
A_23_P64173	-2.8738	2.0805	-0.7933
A_23_P378526	-1.815	2.574	0.759
A_24_P291426	-3.0932	2.7715	-0.3217
A_32_P224234	-0.8372	2.1495	1.3123
A_23_P397347	-2.5977	2.5015	-0.0962
A_23_P309739	-0.8583	2.1154	1.2571
A_23_P120899	-1.3018	2.7147	1.4129
A_32_P122579	0.6126	2.8956	3.5082
A_23_P426292	-1.5019	2.7476	1.2457
A_24_P472055	-3.2276	2.9147	-0.3129
A_24_P59899	-1.0271	2.2193	1.1922
A_24_P798709	-3.0812	3.1157	0.0345
A_24_P262201	-3.2114	2.2854	-0.926
A_24_P565908	-0.9116	2.65	1.7384
A_32_P55438	-0.7223	2.1052	1.3829
A_23_P122216	-2.9359	2.9411	0.0052
A_24_P337397	-2.8944	2.7115	-0.1829
A_24_P132019	-1.6932	2.0398	0.3466
A_23_P89155	-1.212	2.4377	1.2257
A_32_P12562	-2.7512	2.0276	-0.7236
A_32_P198029	-2.2024	2.4827	0.2803
A_24_P191664	-2.5537	2.2455	-0.3082
A_24_P40978	-1.9569	4.9494	2.9925
A_23_P217611	-2.5126	2.463	-0.0496
A_32_P19193	-2.2334	5.5567	3.3233
A_23_P328729	-3.0277	2.7465	-0.2812
A_23_P371966	-2.7638	3.0017	0.2379
A_23_P92499	0.3371	2.2384	2.5755
A_23_P131240	-2.495	4.5778	2.0828
A_23_P252283	-1.086	2.1404	1.0544
A_23_P97923	0.3775	2.0609	2.4384
A_32_P4433	-0.8786	2.6889	1.8103
A_23_P60306	-1.43	3.1607	1.7307
A_24_P108262	-2.3559	3.0275	0.6716
A_32_P188178	-1.2065	2.7781	1.5716
A_24_P943957	-1.3555	2.6665	1.311
A_24_P107336	-2.4053	2.0396	-0.3657

A_23_P212360	-2.5493	3.3784	0.8291
A_32_P15464	-2.4985	2.8951	0.3966
A_23_P215296	-1.4803	2.9501	1.4698
A_23_P161719	-2.6808	2.7299	0.0491
A_23_P17192	-2.6464	3.9865	1.3401
A_24_P196851	-3.5843	2.0983	-1.486
A_24_P826348	-4.4214	3.1279	-1.2935
A_23_P39971	-1.592	2.0704	0.4784
A_32_P409919	-1.9337	2.297	0.3633
A_24_P485219	-2.8606	2.0048	-0.8558
A_23_P404893	-0.8653	2.006	1.1407
A_32_P190682	-4.5588	3.1978	-1.361
A_24_P598836	1.5009	2.4514	3.9523
A_23_P16354	-0.7609	2.799	2.0381
A_32_P98940	-2.9393	2.3716	-0.5677
A_23_P3823	-2.8434	3.0685	0.2251
A_24_P85942	-1.943	2.2909	0.3479
A_24_P113131	-1.9627	2.3295	0.3668
A_23_P117190	-4.3772	2.1152	-2.262
A_23_P70634	-0.6002	2.9671	2.3669
A_23_P372331	-1.7993	2.3791	0.5798
A_32_P184746	-2.4516	3.1026	0.651
A_24_P406714	-3.002	2.4881	-0.5139
A_32_P115505	-2.8139	2.0655	-0.7484
A_23_P354193	-2.0593	3.4694	1.4101
A_23_P153783	-3.0277	2.2711	-0.7566
A_32_P224638	-2.8875	2.2905	-0.597
A_23_P88554	-1.4979	2.0485	0.5506
A_23_P432610	-0.9997	3.349	2.3493
A_24_P145633	-4.0062	2.7557	-1.2505
A_24_P747443	-0.2984	2.6184	2.32
A_24_P557479	-1.4598	2.1564	0.6966
A_32_P201979	-1.7319	2.3007	0.5688
A_24_P346431	-2.0521	2.2017	0.1496
A_24_P253827	-1.109	2.123	1.014
A_23_P100754	-2.0409	2.3504	0.3095
A_23_P144639	-2.493	2.0586	-0.4344
A_23_P58251	-2.2199	2.6335	0.4136
A_24_P607107	-1.7616	2.3616	0.6
A_24_P403168	-1.795	3.4046	1.6096
A_23_P71530	-2.9465	2.9412	-0.0053
A_23_P15226	-0.1308	3.8485	3.7177
A_24_P634530	-2.9918	2.0372	-0.9546
A_24_P307896	-3.0044	2.6496	-0.3548
A_24_P95439	-1.5136	2.3918	0.8782
A_24_P919668	1.6829	2.2766	3.9595
A_23_P371787	-3.0779	2.4543	-0.6236
A_24_P918317	-2.3078	2.4615	0.1537
A_23_P27048	-1.8876	2.0069	0.1193
A_23_P210224	-2.8371	2.1827	-0.6544
A_23_P41854	-0.7053	2.0393	1.334
A_23_P132378	-1.974	2.3223	0.3483

A_23_P24616	-0.6804	2.768	2.0876
A_23_P163258	-2.9977	2.0898	-0.9079
A_23_P377376	0.6482	2.1329	2.7811
A_23_P113111	-1.7102	2.9613	1.2511
A_23_P165783	-3.4121	2.6141	-0.798
A_23_P1505	-5.0275	3.8059	-1.2216
A_23_P55731	-2.1115	3.065	0.9535
A_23_P360964	-1.8434	2.6547	0.8113
A_23_P18123	-3.4476	2.148	-1.2996
A_24_P887857	-1.5511	2.3525	0.8014
A_23_P385105	-1.5559	2.601	1.0451
A_23_P126075	-1.3989	2.0705	0.6716
A_23_P102508	-2.2415	2.9884	0.7469
A_24_P225961	-0.1668	2.5311	2.3643
A_23_P122216	-3.6721	2.3348	-1.3373
A_23_P165343	-2.2958	2.6348	0.339
A_24_P159548	-0.9296	2.0954	1.1658
A_23_P422831	-0.8419	2.619	1.7771
A_32_P77989	-1.7489	3.1774	1.4285
A_23_P103062	-1.7306	2.0561	0.3255
A_24_P89038	-2.5341	2.1093	-0.4248
A_23_P376188	-0.6552	2.2856	1.6304
A_24_P32085	-4.0209	2.1101	-1.9108
A_32_P169316	-0.5939	2.72	2.1261
A_23_P82913	-2.6074	3.2896	0.6822
A_24_P288979	-1.383	2.2767	0.8937
A_23_P18901	-4.7712	2.8041	-1.9671
A_24_P318836	-1.5972	2.1507	0.5535
A_23_P351844	-3.2106	2.0825	-1.1281
A_23_P97892	-2.7571	2.6804	-0.0767
A_24_P64918	-1.843	2.5671	0.7241
A_23_P145644	-3.0254	2.8869	-0.1385
A_23_P211136	-1.2745	2.483	1.2085
A_23_P101623	-2.1303	2.0249	-0.1054
A_23_P252928	-4.7443	2.8943	-1.85
A_23_P132277	-4.7255	3.5619	-1.1636
A_24_P74753	-0.494	2.5673	2.0733
A_24_P339664	-1.8015	2.7491	0.9476
A_32_P171232	-2.6108	2.1874	-0.4234
A_24_P119141	-0.2214	2.5669	2.3455
A_23_P411321	-1.3446	2.2214	0.8768
A_24_P49421	0.2051	2.4282	2.6333
A_23_P109322	-1.8017	2.8921	1.0904
A_23_P204640	-4.0766	2.2982	-1.7784
A_23_P135157	-1.2285	2.136	0.9075
A_24_P156288	-3.0642	2.4207	-0.6435
A_23_P502320	-1.5777	2.2588	0.6811
A_24_P315921	-3.6616	2.0417	-1.6199
A_23_P137196	-1.3207	2.0154	0.6947
A_24_P519504	-2.4377	2.1179	-0.3198
A_24_P140204	-7.3953	4.8645	-2.5308
A_23_P42065	-2.8123	3.7857	0.9734

A_23_P123763	-1.1004	2.249	1.1486
A_32_P107876	-1.0842	2.0844	1.0002
A_32_P160537	2.0805	2.5832	4.6637
A_23_P77440	-2.7784	2.6013	-0.1771
A_24_P235131	-1.417	2.1879	0.7709
A_24_P306214	-2.5216	3.946	1.4244
A_32_P211558	-1.2665	2.4949	1.2284
A_32_P175349	-1.8288	2.9656	1.1368
A_23_P84320	-1.8188	2.7132	0.8944
A_23_P339309	0.2281	2.5611	2.7892
A_23_P85201	-1.6852	3.1951	1.5099
A_24_P110601	-3.9476	2.2543	-1.6933
A_23_P54100	-1.5936	2.0401	0.4465
A_23_P138492	-1.5314	2.1712	0.6398
A_24_P314351	-1.8358	3.0509	1.2151
A_23_P207367	-2.2092	2.8047	0.5955
A_32_P195137	3.2339	2.1932	5.4271
A_24_P38387	-3.0463	2.8995	-0.1468
A_23_P391479	-1.8757	4.4616	2.5859
A_23_P344673	-0.3643	2.4593	2.095
A_23_P214273	-1.5052	2.5293	1.0241
A_32_P204330	-2.6818	2.795	0.1132
A_23_P206441	-2.1401	2.5207	0.3806
A_23_P59869	-2.0219	2.0458	0.0239
A_23_P147514	-2.9129	2.2794	-0.6335
A_23_P62752	-1.794	2.4639	0.6699
A_23_P156953	-1.7175	2.32	0.6025
A_32_P220938	-0.8894	2.7929	1.9035
A_23_P139682	-2.3267	2.1465	-0.1802
A_32_P199292	-2.3102	2.0182	-0.292
A_23_P357101	-2.4554	2.1832	-0.2722
A_32_P42253	-2.7829	2.196	-0.5869
A_24_P32920	-1.5868	2.0132	0.4264
A_23_P401238	-2.1647	3.4221	1.2574
A_24_P269619	-2.5298	2.7001	0.1703
A_32_P128097	-1.1724	3.2294	2.057
A_23_P156620	-0.8806	2.0296	1.149
A_24_P381838	-1.9466	2.3712	0.4246
A_32_P30834	-0.9065	3.5686	2.6621
A_32_P15320	1.9852	2.2422	4.2274
A_24_P342150	-2.896	2.4353	-0.4607
A_23_P394836	-1.254	2.1673	0.9133
A_23_P130764	-2.7679	2.4099	-0.358
A_23_P257003	-2.7349	2.887	0.1521
A_23_P314250	-7.4075	5.0638	-2.3437
A_23_P123362	-1.8528	2.9473	1.0945
A_24_P145216	-0.8068	2.0159	1.2091
A_23_P314811	-2.5147	2.1121	-0.4026
A_32_P137604	-2.3203	2.7265	0.4062
A_24_P135444	-1.6226	3.0999	1.4773
A_32_P148407	-1.1077	2.0301	0.9224
A_23_P310421	-2.4796	2.2079	-0.2717

A_23_P361773	-2.9096	2.5475	-0.3621
A_23_P205216	-1.5319	2.3747	0.8428
A_24_P173566	-1.042	2.1433	1.1013
A_24_P735072	-2.4278	2.4645	0.0367
A_23_P33607	-0.0879	3.0997	3.0118
A_23_P18559	-0.9624	2.5703	1.6079
A_23_P136870	-1.7484	2.5942	0.8458
A_23_P89931	-1.1071	2.0974	0.9903
A_23_P313828	-2.0071	3.069	1.0619
A_23_P30474	0.311	2.3274	2.6384
A_23_P258048	-3.2986	2.3923	-0.9063
A_23_P6335	-3.3503	2.2525	-1.0978
A_23_P18266	-0.2403	2.3621	2.1218
A_24_P928038	-3.2436	2.6426	-0.601
A_24_P161827	-1.3027	2.1451	0.8424
A_23_P208925	-2.9189	2.1918	-0.7271
A_23_P129602	-3.1023	3.2728	0.1705
A_24_P913016	-3.6723	2.8491	-0.8232
A_23_P218058	-2.4561	2.1008	-0.3553
A_24_P732099	0.2368	2.6077	2.8445
A_23_P301896	-1.7863	2.4056	0.6193
A_23_P303087	-3.0347	2.4525	-0.5822
A_24_P406814	-1.2233	2.7556	1.5323
A_23_P131337	-2.1774	2.309	0.1316
A_24_P256243	-0.9315	2.3698	1.4383
A_23_P218937	-0.2546	2.7827	2.5281
A_23_P1038	-1.5864	2.1486	0.5622
A_32_P148507	-2.5629	2.1036	-0.4593
A_23_P72961	-0.3594	2.1516	1.7922
A_24_P345131	-3.1178	2.1523	-0.9655
A_23_P2066	-0.7879	2.5123	1.7244
A_32_P216030	-1.5755	2.2526	0.6771
A_23_P380526	-3.3331	2.4084	-0.9247
A_23_P85024	-2.73	3.0954	0.3654
A_24_P216968	-0.8606	2.1425	1.2819
A_32_P790284	-1.0435	2.3218	1.2783
A_24_P24060	-2.5966	4.075	1.4784
A_23_P112481	-1.7615	2.2033	0.4418
A_23_P36322	-2.04	2.4798	0.4398
A_24_P494501	-2.4757	2.3203	-0.1554
A_32_P143000	-2.6746	2.4234	-0.2512
A_23_P259851	-1.6935	2.0943	0.4008
A_32_P123088	-2.234	2.2174	-0.0166
A_23_P414793	-3.5968	2.2797	-1.3171
A_23_P214011	-1.9233	2.4088	0.4855
A_32_P101039	-2.4676	3.0653	0.5977
A_23_P18903	-1.2587	2.1323	0.8736
A_23_P204484	-1.3044	2.9478	1.6434
A_23_P401580	-0.1196	4.0128	3.8932
A_32_P125135	-0.4538	2.1657	1.7119
A_23_P207445	-0.0022	2.3398	2.3376
A_23_P169233	-2.3102	2.2223	-0.0879

A_32_P83000	-1.9862	2.0527	0.0665
A_23_P3502	-2.0668	2.2615	0.1947
A_23_P27265	-1.8121	2.1017	0.2896
A_32_P214503	-3.5111	2.2624	-1.2487
A_24_P190472	-2.8956	3.5004	0.6048
A_24_P21056	-1.573	2.2867	0.7137
A_23_P144438	-3.7708	2.2597	-1.5111
A_23_P315892	-2.9421	2.327	-0.6151
A_32_P105730	-1.7308	2.7667	1.0359
A_23_P500936	-1.9692	2.1299	0.1607
A_23_P500614	-1.9004	3.3963	1.4959
A_23_P50646	-0.5676	2.0892	1.5216
A_23_P333330	-1.8053	2.671	0.8657
A_32_P453321	-2.7612	2.3786	-0.3826
A_32_P93045	-1.8602	2.4574	0.5972
A_23_P108673	0.6908	2.0374	2.7282
A_23_P404821	-2.8312	2.1151	-0.7161
A_24_P392109	-1.1864	2.3489	1.1625
A_24_P92823	-2.2004	3.2691	1.0687
A_23_P122216	-7.2587	6.168	-1.0907
A_23_P102575	-2.9205	2.3532	-0.5673
A_23_P210763	-2.7955	2.9123	0.1168
A_23_P69242	-3.0494	3.475	0.4256
A_23_P215275	-1.9407	2.211	0.2703
A_23_P94118	-0.3081	2.4476	2.1395
A_24_P255645	-2.9874	3.1991	0.2117
A_23_P216071	-0.009	2.0075	1.9985
A_23_P166336	-3.2364	3.0061	-0.2303
A_23_P250813	-7.4051	7.0516	-0.3535
A_32_P127501	-4.7201	4.4576	-0.2625
A_24_P138713	-1.9909	2.3622	0.3713
A_24_P924261	-7.2521	3.9012	-3.3509
A_24_P34568	-2.7048	2.1421	-0.5627
A_23_P204998	-3.3196	2.9491	-0.3705
A_24_P269598	-2.4403	2.1222	-0.3181
A_24_P922838	-2.5566	3.4441	0.8875
A_23_P136552	-2.2826	2.8628	0.5802
A_32_P204722	-3.1785	2.3638	-0.8147
A_24_P81900	-2.1878	3.1582	0.9704
A_23_P15090	-1.4013	3.1858	1.7845
A_24_P15973	-7.2676	4.7958	-2.4718
A_23_P124486	-1.7196	2.7251	1.0055
A_24_P270376	-1.0602	2.652	1.5918
A_23_P308073	-2.3895	2.0148	-0.3747
A_32_P222521	-1.1237	2.3698	1.2461
A_23_P156826	-3.0349	3.3375	0.3026
A_23_P67771	-1.9991	2.7701	0.771
A_23_P95070	-2.3643	2.8871	0.5228
A_23_P161769	-3.4962	2.7547	-0.7415
A_23_P162782	-1.6293	2.1419	0.5126
A_24_P289709	-1.2578	2.3708	1.113
A_23_P66473	-1.341	2.1117	0.7707

A_32_P139505	-2.8916	2.3429	-0.5487
A_23_P124585	-2.9302	2.7397	-0.1905
A_24_P208794	-2.5073	2.0497	-0.4576
A_23_P38085	-2.2575	2.1523	-0.1052
A_24_P12539	-2.7333	2.1424	-0.5909
A_23_P162374	-2.3345	3.8608	1.5263
A_23_P51966	-1.8613	2.2716	0.4103
A_32_P204381	-2.6775	2.3117	-0.3658
A_23_P342910	-1.8343	2.5266	0.6923
A_24_P263259	-1.2364	2.3239	1.0875
A_23_P193	-1.6242	2.6389	1.0147
A_23_P21882	-2.1064	3.0261	0.9197
A_23_P38365	-2.2956	2.3959	0.1003
A_23_P30736	-2.4033	3.2065	0.8032
A_23_P354170	-1.4337	2.1097	0.676
A_23_P40455	-0.3513	2.0171	1.6658
A_32_P71032	-2.3198	2.7167	0.3969
A_23_P214658	-2.7984	2.2137	-0.5847
A_32_P190036	-1.4182	3.0169	1.5987
A_32_P185393	-3.0372	2.3998	-0.6374
A_23_P71926	-1.6958	2.2444	0.5486
A_23_P106174	-2.2249	2.8252	0.6003
A_24_P69095	-2.6061	2.9161	0.31
A_24_P384969	-1.676	4.8003	3.1243
A_23_P68198	-1.8677	3.7271	1.8594
A_23_P123234	-2.4159	2.141	-0.2749
A_24_P282043	-1.3868	3.6243	2.2375
A_32_P147969	-0.9891	2.7953	1.8062
A_23_P136961	-0.8338	2.2413	1.4075
A_23_P71537	-1.923	2.523	0.6
A_32_P130522	-0.6823	3.4578	2.7755
A_23_P155009	-2.3622	2.4429	0.0807
A_23_P205370	-1.3064	2.4087	1.1023
A_23_P385851	-1.6075	2.1144	0.5069
A_23_P319557	-2.2428	4.2009	1.9581
A_23_P54147	-1.3147	2.1564	0.8417
A_23_P27279	-1.1692	2.0203	0.8511
A_24_P44931	-2.2546	2.6363	0.3817
A_24_P923483	-1.3811	2.5322	1.1511
A_24_P531312	-2.5657	2.375	-0.1907
A_24_P370201	-1.7219	2.7245	1.0026
A_23_P125001	-0.4863	2.3195	1.8332
A_32_P97513	-1.0486	2.1904	1.1418
A_23_P149818	-1.1504	2.0905	0.9401
A_24_P304760	-1.9964	2.1984	0.202
A_32_P14721	-1.2122	3.0401	1.8279
A_23_P157620	-2.4576	2.7381	0.2805
A_23_P129174	-2.1668	3.1836	1.0168
A_23_P146325	-2.8106	2.0663	-0.7443
A_24_P728115	-0.9636	2.9398	1.9762
A_23_P106806	-1.8258	2.2546	0.4288
A_23_P116578	-1.1056	2.2763	1.1707

A_24_P404628	-2.629	3.9245	1.2955
A_23_P216556	-2.1021	2.6531	0.551
A_24_P870463	-1.0905	2.9501	1.8596
A_24_P556318	-1.5174	3.3683	1.8509
A_24_P70888	-2.3718	2.2386	-0.1332
A_24_P919916	-1.7055	7.5659	5.8604
A_23_P384405	-1.4005	2.1135	0.713
A_23_P109655	-0.7361	2.1045	1.3684
A_23_P216806	-2.7596	2.0967	-0.6629
A_23_P3424	-2.3376	2.2232	-0.1144
A_32_P47200	-1.9464	3.15	1.2036
A_23_P68511	-2.3109	2.0929	-0.218
A_24_P931598	-2.4972	2.945	0.4478
A_23_P39871	-2.7099	2.2106	-0.4993
A_24_P222165	-2.2642	2.6246	0.3604
A_24_P759674	-1.5706	4.7534	3.1828
A_23_P163455	-2.1263	2.3791	0.2528
A_23_P254353	-1.2207	2.5583	1.3376
A_32_P134772	-2.2163	2.1805	-0.0358
A_24_P272761	-2.4962	2.2044	-0.2918
A_23_P112846	-0.3299	2.0258	1.6959
A_23_P123315	0.4881	2.0575	2.5456
A_32_P193939	-0.8456	3.8417	2.9961
A_23_P305321	-2.3498	2.6497	0.2999
A_24_P401921	-2.1895	2.267	0.0775
A_24_P795594	-2.3958	2.2629	-0.1329
A_23_P115046	-2.59	2.4959	-0.0941
A_24_P925274	-1.0955	2.4184	1.3229
A_32_P202977	-2.8087	2.6321	-0.1766
A_23_P21457	-0.0633	2.0777	2.0144
A_24_P925124	-1.1233	2.2517	1.1284
A_24_P401686	-1.7645	2.7103	0.9458
A_24_P265346	-2.8338	2.6956	-0.1382
A_32_P123788	-2.5072	2.5121	0.0049
A_32_P5150	-2.2784	2.3024	0.024
A_32_P155416	-1.9572	2.0617	0.1045
A_24_P221323	-2.6137	2.4061	-0.2076
A_23_P333420	-2.1025	2.518	0.4155
A_23_P54469	-1.5567	2.7775	1.2208
A_24_P44891	-2.6069	4.0677	1.4608
A_32_P33304	-2.023	2.8581	0.8351
A_24_P889103	-2.4632	2.8324	0.3692
A_23_P251002	-1.3658	2.1659	0.8001
A_24_P103744	-1.6628	2.271	0.6082
A_23_P77223	-1.0517	2.953	1.9013
A_24_P357709	-2.5627	3.2506	0.6879
A_24_P326491	-0.4585	2.8818	2.4233
A_32_P209148	-1.6987	2.9905	1.2918
A_23_P23947	-1.2674	2.3634	1.096
A_23_P167159	-0.8512	5.5067	4.6555
A_23_P169017	0.4129	2.2005	2.6134
A_24_P263310	-0.9721	5.1337	4.1616

A_32_P234591	-0.992	2.4311	1.4391
A_24_P391586	-1.2954	2.2063	0.9109
A_23_P18152	-1.7123	2.0777	0.3654
A_24_P162226	-2.8348	2.0092	-0.8256
A_23_P52109	-0.6372	2.7799	2.1427
A_24_P78862	-2.7099	2.0532	-0.6567
A_32_P113736	-2.2536	2.3317	0.0781
A_24_P204067	-3.0135	4.1532	1.1397
A_23_P84860	-1.1189	2.5008	1.3819
A_24_P929388	-2.5001	2.7064	0.2063
A_32_P50587	-1.4465	2.581	1.1345
A_23_P127339	-1.8102	3.3201	1.5099
A_24_P50972	-1.6545	2.9886	1.3341
A_24_P623782	-1.9896	2.0423	0.0527
A_32_P27479	-2.4068	3.2558	0.849
A_24_P355693	-1.7488	2.0027	0.2539
A_32_P101653	-1.8889	2.8232	0.9343
A_23_P345778	-2.0998	2.1628	0.063
A_24_P698136	-0.4141	2.8326	2.4185
A_23_P109393	-1.7263	2.4049	0.6786

UNIQID	Gene name
WID:6480720 || A_24_P100368 || MAP:Xp11.4 || DYNLT3 -- dynein, light c	WID:6480720
WID:6485103 || A_23_P375147 || MAP: || -- MRNA; cDNA DKFZp686	WID:6485103
WID:6515818 || A_23_P40952 || MAP:3p25.1 || RAF1 -- v-raf-1 murine le	WID:6515818
WID:6475693 || A_32_P201564 || MAP:17q25.1 || CD300E -- CD300e mol	WID:6475693
WID:6494148 || A_23_P348257 || MAP:12q23.3 || NUAK1 -- NUAK family,	WID:6494148
WID:6510419 || A_24_P938352 || MAP:12q15 || CPM -- carboxypeptidase	WID:6510419
WID:6489246 || A_24_P917123 || MAP:6p22.3 || MYLIP -- myosin regulato	WID:6489246
WID:6506374 || A_23_P153070 || MAP:17q23.2 || BCAS3 -- breast carcino	WID:6506374
WID:6497827 || A_24_P900730 || MAP: || -- Unknown ||	WID:6497827
WID:6585756 || A_23_P70328 || MAP:6p12.3 || CENPQ -- centromere pro	WID:6585756
WID:6585761 || A_23_P6335 || MAP:22q11.21 || SERPIND1 -- serpin pep	WID:6585761
WID:6492000 || A_24_P118489 || MAP:15q11.2 || WHAMML1 -- WAS prot	WID:6492000
WID:6497608 || A_24_P124325 || MAP: || IKZF5 -- IKAROS family zinc	WID:6497608
WID:6505633 || A_32_P6233 || MAP: || -- Transcribed locus ||	WID:6505633
WID:6482066 || A_23_P64173 || MAP:11q22.3 || CARD16 -- caspase recr	WID:6482066
WID:6479145 || A_23_P378526 || MAP:20q13.33 || RTEL1 -- regulator of te	WID:6479145
WID:6514902 || A_24_P291426 || MAP:2q11.2 || REV1 -- REV1 homolog	WID:6514902
WID:6513086 || A_32_P224234 || MAP:1p31.3 || LOC645195 -- PREDICT	WID:6513086
WID:6488987 || A_23_P397347 || MAP:6q22.31 || MCM9 -- minichromoson	WID:6488987
WID:6501323 || A_23_P309739 || MAP:6q25.1 || ESR1 -- estrogen recepto	WID:6501323
WID:6487384 || A_23_P120899 || MAP:22q12.3 || CSF2RB -- colony stimu	WID:6487384
WID:6480399 || A_32_P122579 || MAP: || EZH2 -- Enhancer of zeste I	WID:6480399
WID:6489611 || A_23_P426292 || MAP:6p21.31 || MAPK14 -- mitogen-acti	WID:6489611
WID:6482853 || A_24_P472055 || MAP: || -- Transcribed locus ||	WID:6482853
WID:6516270 || A_24_P59899 || MAP: || LAMA1 -- Laminin, alpha 1 ||	WID:6516270
WID:6478065 || A_24_P798709 || MAP: || NPAL2 -- NIPA-like domain	WID:6478065
WID:6493745 || A_24_P262201 || MAP: || SULT1A3 -- sulfotransferase	WID:6493745
WID:6500310 || A_24_P565908 || MAP: || -- Unknown ||	WID:6500310
WID:6492118 || A_32_P55438 || MAP: || -- Transcribed locus ||	WID:6492118
WID:6586072 || A_23_P122216 || MAP:5q23.1 || LOX -- lysyl oxidase (LO	WID:6586072
WID:6506899 || A_24_P337397 || MAP:5q13.2 || ANKRA2 -- ankyrin repea	WID:6506899
WID:6485703 || A_24_P132019 || MAP: || LNPEP -- Leucyl/cystinyl am	WID:6485703
WID:6507479 || A_23_P89155 || MAP:17q25.1 || CDK3 -- cyclin-dependen	WID:6507479
WID:6498109 || A_32_P12562 || MAP: || -- CDNA clone IMAGE:4620	WID:6498109
WID:6502550 || A_32_P198029 || MAP: || -- Transcribed locus ||	WID:6502550
WID:6478127 || A_24_P191664 || MAP:3q26.2 || GOLIM4 -- golgi integral	WID:6478127
WID:6497049 || A_24_P40978 || MAP:8q24.3 || CYHR1 -- cysteine/histidi	WID:6497049
WID:6495980 || A_23_P217611 || MAP:Xq22.1 || ARMCX3 -- armadillo rep	WID:6495980
WID:6497966 || A_32_P19193 || MAP: || -- Unknown ||	WID:6497966
WID:6476144 || A_23_P328729 || MAP:4q22.1 || KLHL8 -- kelch-like 8 (Dr	WID:6476144
WID:6497764 || A_23_P371966 || MAP: || FAM171B -- Family with sec	WID:6497764
WID:6486692 || A_23_P92499 || MAP:4q31.3 || TLR2 -- toll-like receptor 2	WID:6486692
WID:6475844 || A_23_P131240 || MAP:2p23.3 || UBXN2A -- UBX domain	WID:6475844
WID:6475549 || A_23_P252283 || MAP:17q11.2 || RNF135 -- ring finger pr	WID:6475549
WID:6517113 || A_23_P97923 || MAP:10q21.1 || PHYHIPL -- phytanoyl-Co	WID:6517113
WID:6501278 || A_32_P4433 || MAP: || -- Transcribed locus ||	WID:6501278
WID:6502707 || A_23_P60306 || MAP:9q33.1 || TLR4 -- toll-like receptor 4	WID:6502707
WID:6487931 || A_24_P108262 || MAP:17q25.1 || SDK2 -- sidekick homolo	WID:6487931
WID:6479161 || A_32_P188178 || MAP:2q36.3 || TRIP12 -- thyroid hormon	WID:6479161
WID:6494331 || A_24_P943957 || MAP:2q33.3 || PIP5K3 -- phosphatidylin	WID:6494331
WID:6484999 || A_24_P107336 || MAP:11q25 || VPS26B -- vacuolar prote	WID:6484999

WID:6497539 || A_23_P212360 || MAP: || CCR9 -- Chemokine (C-C m WID:6497539
WID:6486552 || A_32_P15464 || MAP:8q12.1 || TMEM68 -- transmembra WID:6486552
WID:6499148 || A_23_P215296 || MAP:7p14.1 || CDC2L5 -- cell division c WID:6499148
WID:6493099 || A_23_P161719 || MAP:11q22.3 || CWF19L2 -- CWF19-like WID:6493099
WID:6481555 || A_23_P17192 || MAP:2q31.1 || RAPGEF4 -- Rap guanine WID:6481555
WID:6481250 || A_24_P196851 || MAP:9p13.3 || TLN1 -- talin 1 (TLN1), m WID:6481250
WID:6484813 || A_24_P826348 || MAP:2q13 || ZC3H6 -- zinc finger CCC WID:6484813
WID:6500307 || A_23_P39971 || MAP:2p16.2 || C2orf30 -- chromosome 2 WID:6500307
WID:6476513 || A_32_P409919 || MAP:7q11.23 || PHTF2 -- putative home WID:6476513
WID:6474974 || A_24_P485219 || MAP:Xq21.1 || MAGT1 -- magnesium tr WID:6474974
WID:6503661 || A_23_P404893 || MAP:16q24.3 || AFG3L1 -- AFG3 ATPas WID:6503661
WID:6501889 || A_32_P190682 || MAP: || -- Unknown || WID:6501889
WID:6517296 || A_24_P598836 || MAP:10p11.22 || ITGB1 -- integrin, beta 1 WID:6517296
WID:6505912 || A_23_P16354 || MAP:19q13.12 || ZNF382 -- zinc finger pr WID:6505912
WID:6491750 || A_32_P98940 || MAP: || -- Unknown || WID:6491750
WID:6480455 || A_23_P3823 || MAP:16p11.2 || BCKDK -- branched chain WID:6480455
WID:6509725 || A_24_P85942 || MAP:2q12.3 || GCC2 -- GRIP and coiled WID:6509725
WID:6493122 || A_24_P113131 || MAP:17q22 || BZRAP1 -- benzodiazapi WID:6493122
WID:6500126 || A_23_P117190 || MAP:13q14.11 || DNAJC15 -- DnaJ (Hsp WID:6500126
WID:6499449 || A_23_P70634 || MAP:6q24.1 || NMBR -- neuromedin B re WID:6499449
WID:6495860 || A_23_P372331 || MAP:3p26.3 || TRNT1 -- tRNA nucleotic WID:6495860
WID:6489926 || A_32_P184746 || MAP: || -- Transcribed locus || WID:6489926
WID:6487107 || A_24_P406714 || MAP:10p12.31 || MLLT10 -- myeloid/lymp WID:6487107
WID:6484163 || A_32_P115505 || MAP:21q21.3 || RNF160 -- ring finger pr WID:6484163
WID:6496613 || A_23_P354193 || MAP:20p13 || TBC1D20 -- TBC1 doma WID:6496613
WID:6517988 || A_23_P153783 || MAP:19q13.12 || USF2 -- upstream trans WID:6517988
WID:6506799 || A_32_P224638 || MAP: || -- Transcribed locus || WID:6506799
WID:6506394 || A_23_P88554 || MAP: || ADAMTS17 -- ADAM metallo WID:6506394
WID:6484876 || A_23_P432610 || MAP:16q12.1 || N4BP1 -- NEDD4 bindin WID:6484876
WID:6495771 || A_24_P145633 || MAP:1q32.1 || GPR37L1 -- G protein-co WID:6495771
WID:6501297 || A_24_P747443 || MAP: || -- Transcribed locus || WID:6501297
WID:6503155 || A_24_P557479 || MAP:17p13.2 || XAF1 -- XIAP associate WID:6503155
WID:6515721 || A_32_P201979 || MAP:10q23.32 || HECTD2 -- HECT doma WID:6515721
WID:6476559 || A_24_P346431 || MAP:7p12.3 || TNS3 -- tensin 3 (TNS3) WID:6476559
WID:6485544 || A_24_P253827 || MAP:17q12 || AP2B1 -- adaptor-related WID:6485544
WID:6492701 || A_23_P100754 || MAP:17q24.1 || SMURF2 -- SMAD spec WID:6492701
WID:6515556 || A_23_P144639 || MAP: || TMCO6 -- Transmembrane WID:6515556
WID:6515634 || A_23_P58251 || MAP:4p16.1 || CPZ -- carboxypeptidase WID:6515634
WID:6511317 || A_24_P607107 || MAP: || -- Unknown || WID:6511317
WID:6490567 || A_24_P403168 || MAP:6p25.2 || PRPF4B -- PRP4 pre-mR WID:6490567
WID:6586506 || A_23_P71530 || MAP:8q24.12 || TNFRSF11B -- tumor ne WID:6586506
WID:6586520 || A_23_P15226 || MAP: || -- Unknown || WID:6586520
WID:6502100 || A_24_P634530 || MAP:16p13.12 || FLJ11151 -- hypothetica WID:6502100
WID:6513277 || A_24_P307896 || MAP: || GIGYF2 -- GRB10 interactin WID:6513277
WID:6486291 || A_24_P95439 || MAP:11p15.4 || CARS -- cysteinyl-tRNA s WID:6486291
WID:6497260 || A_24_P919668 || MAP: || NAPG -- N-ethylmaleimide-s WID:6497260
WID:6488161 || A_23_P371787 || MAP:14q24.1 || KIAA0247 -- KIAA0247 (WID:6488161
WID:6477824 || A_24_P918317 || MAP:11p15.3 || DKK3 -- dickkopf homol WID:6477824
WID:6483704 || A_23_P27048 || MAP:17q11.2 || TMEM199 -- transmemb WID:6483704
WID:6516824 || A_23_P210224 || MAP:2p23.3 || DPYSL5 -- dihydropyrimi WID:6516824
WID:6479052 || A_23_P41854 || MAP:5p13.1 || CARD6 -- caspase recrui WID:6479052
WID:6495211 || A_23_P132378 || MAP:22q13.31 || CELSR1 -- cadherin, EG WID:6495211

WID:6489937	|| A_23_P24616	|| MAP:11q24.2	|| SIAE -- sialic acid acetyl	WID:6489937
WID:6509743	|| A_23_P163258	|| MAP:15q23	|| PARP6 -- poly (ADP-ribo	WID:6509743
WID:6482919	|| A_23_P377376	|| MAP:2p14	|| ACTR2 -- ARP2 actin-rela	WID:6482919
WID:6488464	|| A_23_P113111	|| MAP:Xq12	|| AR -- androgen receptor	WID:6488464
WID:6513962	|| A_23_P165783	|| MAP:2q37.3	|| MLPH -- melanophilin (M	WID:6513962
WID:6510094	|| A_23_P1505	|| MAP:11q13.2	|| LRP5 -- low density lipop	WID:6510094
WID:6503058	|| A_23_P55731	|| MAP:19q13.2	|| CIC -- capicua homolog (	WID:6503058
WID:6502295	|| A_23_P360964	|| MAP:19q13.32	|| DACT3 -- dapper, antag	WID:6502295
WID:6498302	|| A_23_P18123	|| MAP:3q26.31	|| NLGN1 -- neuroligin 1 (N	WID:6498302
WID:6496766	|| A_24_P887857	|| MAP:17q11.2	|| KRT17P3 -- PREDICTE	WID:6496766
WID:6495998	|| A_23_P385105	|| MAP:2q35	|| PLCD4 -- phospholipase	WID:6495998
WID:6495592	|| A_23_P126075	|| MAP:1q42.2	|| KCNK1 -- potassium cha	WID:6495592
WID:6492737	|| A_23_P102508	|| MAP:2p23.3	|| SLC5A6 -- solute carrier	WID:6492737
WID:6514812	|| A_24_P225961	|| MAP:3p21.31	|| DAG1 -- dystroglycan 1	WID:6514812
WID:6586631	|| A_23_P122216	|| MAP:5q23.1	|| LOX -- lysyl oxidase (LO	WID:6586631
WID:6485628	|| A_23_P165343	|| MAP:2q14.2	|| PTPN4 -- protein tyrosine	WID:6485628
WID:6514781	|| A_24_P159548	|| MAP:17q11.2	|| SLC46A1 -- solute carrie	WID:6514781
WID:6495196	|| A_23_P422831	|| MAP:9q21.11	|| C9orf61 -- chromosome	WID:6495196
WID:6499343	|| A_32_P77989	|| MAP:16q12.1	|| NETO2 -- neuropilin (NR	WID:6499343
WID:6476230	|| A_23_P103062	|| MAP:22q12.2	|| RNF185 -- ring finger pr	WID:6476230
WID:6514725	|| A_24_P89038	|| MAP:4p16.1	|| LOC93622 -- PREDICTE	WID:6514725
WID:6503405	|| A_23_P376188	|| MAP:10p13	|| BEND7 -- BEN domain c	WID:6503405
WID:6505020	|| A_24_P32085	|| MAP:9p21.2	|| MOBKL2B -- MOB1, Mps	WID:6505020
WID:6516129	|| A_32_P169316	|| MAP:	|| -- Transcribed locus ||	WID:6516129
WID:6512493	|| A_23_P82913	|| MAP:8q24.13	|| TRMT12 -- tRNA methylt	WID:6512493
WID:6476997	|| A_24_P288979	|| MAP:12q23.2	|| GNPTAB -- N-acetylgluc	WID:6476997
WID:6508279	|| A_23_P18901	|| MAP:5q22.3	|| ATG12 -- ATG12 autopha	WID:6508279
WID:6514649	|| A_24_P318836	|| MAP:19p13.3	|| FZR1 -- fizzy/cell divisio	WID:6514649
WID:6494290	|| A_23_P351844	|| MAP:	|| CD1B -- CD1b molecule ||	WID:6494290
WID:6510723	|| A_23_P97892	|| MAP:10q24.32	|| LDB1 -- LIM domain bin	WID:6510723
WID:6475682	|| A_24_P64918	|| MAP:14q23.3	|| ZBTB1 -- zinc finger and	WID:6475682
WID:6478662	|| A_23_P145644	|| MAP:7p12.2	|| DDC -- dopa decarboxyla	WID:6478662
WID:6509662	|| A_23_P211136	|| MAP:21q22.2	|| BRWD1 -- bromodomain	WID:6509662
WID:6517573	|| A_23_P101623	|| MAP:19q13.43	|| ZNF667 -- zinc finger pr	WID:6517573
WID:6475338	|| A_23_P252928	|| MAP:Xq28	|| MAGEA12 -- melanoma a	WID:6475338
WID:6497172	|| A_23_P132277	|| MAP:22q12.3	|| MCM5 -- minichromoson	WID:6497172
WID:6481302	|| A_24_P74753	|| MAP:Xq28	|| ATP6AP1 -- ATPase, H+	WID:6481302
WID:6488095	|| A_24_P339664	|| MAP:19p13.3	|| NCLN -- nicalin homolog	WID:6488095
WID:6498176	|| A_32_P171232	|| MAP:	|| -- Transcribed locus ||	WID:6498176
WID:6487266	|| A_24_P119141	|| MAP:	|| PROS1 -- Protein S (alpha)	WID:6487266
WID:6518713	|| A_23_P411321	|| MAP:1q21.3	|| LCE1F -- late cornified e	WID:6518713
WID:6496217	|| A_24_P49421	|| MAP:	|| -- Unknown ||	WID:6496217
WID:6512147	|| A_23_P109322	|| MAP:21q22.2	|| PCP4 -- Purkinje cell pro	WID:6512147
WID:6487751	|| A_23_P204640	|| MAP:12p13.31	|| NANOG -- Nanog home	WID:6487751
WID:6514919	|| A_23_P135157	|| MAP:9p21.2	|| PLAA -- phospholipase A	WID:6514919
WID:6475738	|| A_24_P156288	|| MAP:20p11.21	|| GZF1 -- GDNF-inducibl	WID:6475738
WID:6509851	|| A_23_P502320	|| MAP:16q22.1	|| AGRP -- agouti related p	WID:6509851
WID:6512940	|| A_24_P315921	|| MAP:	|| LOC728448 -- Peptidylproly	WID:6512940
WID:6488748	|| A_23_P137196	|| MAP:Xq24	|| IL13RA1 -- interleukin 13	WID:6488748
WID:6496257	|| A_24_P519504	|| MAP:	|| IGL@ -- Immunoglobulin lar	WID:6496257
WID:6480833	|| A_24_P140204	|| MAP:3p14.3	|| PXK -- PX domain conta	WID:6480833
WID:6484664	|| A_23_P42065	|| MAP:6p12.3	|| TNFRSF21 -- tumor necr	WID:6484664

WID:6496877	A_23_P123763	MAP:9p21.2	C9orf82 -- chromosome	WID:6496877
WID:6488250	A_32_P107876	MAP:4q21.21	FRAS1 -- Fraser syndro	WID:6488250
WID:6492904	A_32_P160537	MAP:	FGF12 -- Fibroblast growth	WID:6492904
WID:6502102	A_23_P77440	MAP:16q22.1	NFATC3 -- nuclear facto	WID:6502102
WID:6501464	A_24_P235131	MAP:7p22.1	RNF216 -- ring finger pro	WID:6501464
WID:6487677	A_24_P306214	MAP:16q24.1	KIAA1609 -- KIAA1609 (	WID:6487677
WID:6474978	A_32_P211558	MAP:	C10orf11 -- Chromosome 1	WID:6474978
WID:6503935	A_32_P175349	MAP:	LOC100128942 -- Hypothet	WID:6503935
WID:6495148	A_23_P84320	MAP:	HMX1 -- H6 family homeobo	WID:6495148
WID:6478677	A_23_P339309	MAP:14q24.3	LIN52 -- lin-52 homolog	WID:6478677
WID:6518423	A_23_P85201	MAP:Xq22.2	PLP1 -- proteolipid protei	WID:6518423
WID:6501519	A_24_P110601	MAP:	-- Unknown ||	WID:6501519
WID:6506930	A_23_P54100	MAP:14q23.2	ESR2 -- estrogen recepto	WID:6506930
WID:6488176	A_23_P138492	MAP:10q24.33	NEURL -- neuralized ho	WID:6488176
WID:6488183	A_24_P314351	MAP:Xq24	ZBTB33 -- zinc finger and	WID:6488183
WID:6481655	A_23_P207367	MAP:17q21.2	STAT5A -- signal transd	WID:6481655
WID:6483333	A_32_P195137	MAP:	RAD21 -- RAD21 homolog (	WID:6483333
WID:6504089	A_24_P38387	MAP:8q24.22	NDRG1 -- N-myc downst	WID:6504089
WID:6517809	A_23_P391479	MAP:12q24.23	KIAA1853 -- KIAA1853	WID:6517809
WID:6487972	A_23_P344673	MAP:	LOC401022 -- Hypothetical	WID:6487972
WID:6480583	A_23_P214273	MAP:	MUT -- Methylmalonyl Coen	WID:6480583
WID:6487210	A_32_P204330	MAP:	-- CDNA FLJ36663 fis, clon	WID:6487210
WID:6586963	A_23_P206441	MAP:16q24.3	FANCA -- Fanconi anem	WID:6586963
WID:6488544	A_23_P59869	MAP:	GJC3 -- Gap junction protein	WID:6488544
WID:6504831	A_23_P147514	MAP:	RHBDD1 -- Rhomboid doma	WID:6504831
WID:6496696	A_23_P62752	MAP:1p36.22	NPPB -- natriuretic peptid	WID:6496696
WID:6517216	A_23_P156953	MAP:6q25.3	IGF2R -- insulin-like grow	WID:6517216
WID:6498993	A_32_P220938	MAP:10q24.33	LOC729020 -- rcRPE (I	WID:6498993
WID:6478871	A_23_P139682	MAP:12p13.31	PZP -- pregnancy-zone	WID:6478871
WID:6506721	A_32_P199292	MAP:18q21.32	LOC728115 -- PREDIC	WID:6506721
WID:6500579	A_23_P357101	MAP:22q13.1	APOBEC3F -- apolipopr	WID:6500579
WID:6498709	A_32_P42253	MAP:	-- Unknown ||	WID:6498709
WID:6518501	A_24_P32920	MAP:	LOC286467 -- Hypothetical p	WID:6518501
WID:6515850	A_23_P401238	MAP:	DDHD2 -- DDHD domain co	WID:6515850
WID:6477541	A_24_P269619	MAP:8q21.3	DECR1 -- 2,4-dienoyl Co	WID:6477541
WID:6492325	A_32_P128097	MAP:	LOC388795 -- Similar to hC	WID:6492325
WID:6488303	A_23_P156620	MAP:6p22.1	ZNF184 -- zinc finger pro	WID:6488303
WID:6510725	A_24_P381838	MAP:15q26.1	MAN2A2 -- mannosidas	WID:6510725
WID:6477788	A_32_P30834	MAP:4q35.2	MGC39584 -- PREDICTE	WID:6477788
WID:6513288	A_32_P15320	MAP:1p21.3	LOC440595 -- PREDICTI	WID:6513288
WID:6493582	A_24_P342150	MAP:15q21.2	LOC100129387 -- hypot	WID:6493582
WID:6480042	A_23_P394836	MAP:14q32.33	INF2 -- inverted formin,	WID:6480042
WID:6475891	A_23_P130764	MAP:19q13.32	KCNJ14 -- potassium ir	WID:6475891
WID:6478982	A_23_P257003	MAP:9q21.13	PCSK5 -- proprotein con	WID:6478982
WID:6502735	A_23_P314250	MAP:9q34.13	FAM78A -- family with se	WID:6502735
WID:6475562	A_23_P123362	MAP:	C8orf17 -- Chromosome 8 c	WID:6475562
WID:6504436	A_24_P145216	MAP:	LOC389906 -- Hypothetical	WID:6504436
WID:6477730	A_23_P314811	MAP:4p12	SLC10A4 -- solute carrier	WID:6477730
WID:6505171	A_32_P137604	MAP:	-- CDNA FLJ13937 fis, clon	WID:6505171
WID:6478968	A_24_P135444	MAP:16q13	AMFR -- autocrine motilit	WID:6478968
WID:6495855	A_32_P148407	MAP:2q21.3	LOC151162 -- hypothetic	WID:6495855
WID:6476737	A_23_P310421	MAP:7p22.2	CHST12 -- carbohydrate	WID:6476737

WID:6481720	|| A_23_P361773	|| MAP:6p21.1	|| CCND3 -- cyclin D3 (CCI	WID:6481720
WID:6474507	|| A_23_P205216	|| MAP:Xq25	|| UTP14A -- UTP14, U3 sr	WID:6474507
WID:6508895	|| A_24_P173566	|| MAP:	|| IGL@ -- Immunoglobulin lar	WID:6508895
WID:6517524	|| A_24_P735072	|| MAP:	|| -- Full length insert cDNA c	WID:6517524
WID:6499563	|| A_23_P33607	|| MAP:17q11.2	|| C17orf42 -- chromosome	WID:6499563
WID:6512529	|| A_23_P18559	|| MAP:4q31.21	|| INPP4B -- inositol polypr	WID:6512529
WID:6587069	|| A_23_P136870	|| MAP:Xq28	|| MAGEA6 -- melanoma ar	WID:6587069
WID:6474959	|| A_23_P89931	|| MAP:19q13.2	|| ZNF574 -- zinc finger prc	WID:6474959
WID:6488755	|| A_23_P313828	|| MAP:17p11.2	|| CENPV -- centromere p	WID:6488755
WID:6511931	|| A_23_P30474	|| MAP:5p13.2	|| WDR70 -- WD repeat doi	WID:6511931
WID:6500215	|| A_23_P258048	|| MAP:	|| -- Transcribed locus ||	WID:6500215
WID:6587113	|| A_23_P6335	|| MAP:22q11.21	|| SERPIND1 -- serpin pep	WID:6587113
WID:6505684	|| A_23_P18266	|| MAP:3p21.31	|| TUSC4 -- tumor suppres	WID:6505684
WID:6504771	|| A_24_P928038	|| MAP:	|| IDE -- Insulin-degrading enz	WID:6504771
WID:6498740	|| A_24_P161827	|| MAP:	|| -- Unknown ||	WID:6498740
WID:6505152	|| A_23_P208925	|| MAP:19p13.3	|| SH3GL1 -- SH3-domain	WID:6505152
WID:6484996	|| A_23_P129602	|| MAP:16p13.3	|| PRSS21 -- protease, se	WID:6484996
WID:6504844	|| A_24_P913016	|| MAP:	|| UROS -- Uroporphyrinogen	WID:6504844
WID:6512571	|| A_23_P218058	|| MAP:12p13.2	|| KLRC4 -- killer cell lectir	WID:6512571
WID:6516838	|| A_24_P732099	|| MAP:11p15.4	|| HBBP1 -- hemoglobin, b	WID:6516838
WID:6487058	|| A_23_P301896	|| MAP:1p36.21	|| PRDM2 -- PR domain cc	WID:6487058
WID:6507213	|| A_23_P303087	|| MAP:7q33	|| PTN -- pleiotrophin (PTN)	WID:6507213
WID:6513900	|| A_24_P406814	|| MAP:10q26.13	|| FAM53B -- family with s	WID:6513900
WID:6490084	|| A_23_P131337	|| MAP:2q35	|| SMARCAL1 -- SWI/SNF	WID:6490084
WID:6475649	|| A_24_P256243	|| MAP:	|| SSX2 -- synovial sarcoma, X	WID:6475649
WID:6503684	|| A_23_P218937	|| MAP:5q31.3	|| PCDHGC3 -- protocadhe	WID:6503684
WID:6517406	|| A_23_P1038	|| MAP:1q32.1	|| JARID1B -- jumonji, AT ric	WID:6517406
WID:6496988	|| A_32_P148507	|| MAP:	|| ADAM15 -- ADAM metallop	WID:6496988
WID:6501940	|| A_23_P72961	|| MAP:	|| PRPS1 -- Phosphoribosyl py	WID:6501940
WID:6494188	|| A_24_P345131	|| MAP:1p22.2	|| LRRC8B -- leucine rich r	WID:6494188
WID:6489700	|| A_23_P2066	|| MAP:11p13	|| APIP -- APAF1 interacting	WID:6489700
WID:6493828	|| A_32_P216030	|| MAP:	|| -- Transcribed locus ||	WID:6493828
WID:6477924	|| A_23_P380526	|| MAP:3q13.13	|| DPPA4 -- developmenta	WID:6477924
WID:6512145	|| A_23_P85024	|| MAP:Xq26.3	|| ARHGEF6 -- Rac/Cdc42	WID:6512145
WID:6483273	|| A_24_P216968	|| MAP:1q32.1	|| NUCKS1 -- nuclear case	WID:6483273
WID:6503540	|| A_32_P790284	|| MAP:18q21.1	|| KATNAL2 -- katanin p60	WID:6503540
WID:6486110	|| A_24_P24060	|| MAP:	|| RHOXF2B -- Rhox homeobc	WID:6486110
WID:6479893	|| A_23_P112481	|| MAP:9p13.3	|| AQP3 -- aquaporin 3 (Gil	WID:6479893
WID:6477288	|| A_23_P36322	|| MAP:11q13.4	|| INPPL1 -- inositol polyph	WID:6477288
WID:6497298	|| A_24_P494501	|| MAP:	|| LOC400236 -- Hypothetical	WID:6497298
WID:6474564	|| A_32_P143000	|| MAP:15q13.1	|| KIAA0574 -- KIAA0574	WID:6474564
WID:6518103	|| A_23_P259851	|| MAP:	|| ADAMTS19 -- ADAM metall	WID:6518103
WID:6501652	|| A_32_P123088	|| MAP:5q22.3	|| TICAM2 -- toll-like recept	WID:6501652
WID:6477048	|| A_23_P414793	|| MAP:3q24	|| CP -- ceruloplasmin (ferro	WID:6477048
WID:6494488	|| A_23_P214011	|| MAP:5p13.3	|| CDH6 -- cadherin 6, type	WID:6494488
WID:6487601	|| A_32_P101039	|| MAP:	|| -- Transcribed locus ||	WID:6487601
WID:6493500	|| A_23_P18903	|| MAP:5q33.3	|| HAVCR2 -- hepatitis A vir	WID:6493500
WID:6489974	|| A_23_P204484	|| MAP:12q24.23	|| RAB35 -- RAB35, mem	WID:6489974
WID:6513268	|| A_23_P401580	|| MAP:5q35.3	|| RMND5B -- required for	WID:6513268
WID:6513200	|| A_32_P125135	|| MAP:15q11.2	|| CXADRP2 -- coxsackie	WID:6513200
WID:6497816	|| A_23_P207445	|| MAP:17q24.3	|| MAP2K6 -- mitogen-acti	WID:6497816
WID:6479400	|| A_23_P169233	|| MAP:9q22.33	|| ZNF510 -- zinc finger pr	WID:6479400

WID:6502711 || A_32_P83000 || MAP:17q21.31 || LOC388387 -- hypotheti WID:6502711
WID:6481841 || A_23_P3502 || MAP:16q24.2 || ZC3H18 -- zinc finger CC WID:6481841
WID:6486896 || A_23_P27265 || MAP:18q22.1 || DSEL -- dermatan sulfat WID:6486896
WID:6506260 || A_32_P214503 || MAP: || -- CDNA clone IMAGE:526 WID:6506260
WID:6488011 || A_24_P190472 || MAP:20q13.12 || SLPI -- secretory leuko WID:6488011
WID:6505959 || A_24_P21056 || MAP:7p21.3 || PHF14 -- PHD finger prot WID:6505959
WID:6478747 || A_23_P144438 || MAP:4p15.32 || C4orf30 -- chromosome WID:6478747
WID:6481039 || A_23_P315892 || MAP:9q34.11 || ST6GALNAC6 -- ST6 (a WID:6481039
WID:6485248 || A_32_P105730 || MAP: || -- Transcribed locus || WID:6485248
WID:6477280 || A_23_P500936 || MAP:20p11.21 || FOXA2 -- forkhead box WID:6477280
WID:6506591 || A_23_P500614 || MAP:1p36.22 || TNFRSF8 -- tumor necr WID:6506591
WID:6482901 || A_23_P50646 || MAP:19q13.31 || LOC390940 -- PREDIC WID:6482901
WID:6486207 || A_23_P333330 || MAP: || ZDHHC24 -- Zinc finger, DH WID:6486207
WID:6501213 || A_32_P453321 || MAP:1p34.1 || NCRNA00082 -- non-pro WID:6501213
WID:6475777 || A_32_P93045 || MAP: || -- MRNA; cDNA DKFZp564 WID:6475777
WID:6496567 || A_23_P108673 || MAP:2p12 || FAM176A -- family with s WID:6496567
WID:6480689 || A_23_P404821 || MAP:7q34 || KIAA1147 -- KIAA1147 (K WID:6480689
WID:6507083 || A_24_P392109 || MAP:16q23.2 || CENPN -- centromere p WID:6507083
WID:6489361 || A_24_P92823 || MAP: || -- Unknown || WID:6489361
WID:6587453 || A_23_P122216 || MAP:5q23.1 || LOX -- lysyl oxidase (LO WID:6587453
WID:6489424 || A_23_P102575 || MAP:20q13.33 || SLC2A4RG -- SLC2A4 WID:6489424
WID:6486716 || A_23_P210763 || MAP:20p12.2 || JAG1 -- jagged 1 (Alagil WID:6486716
WID:6495539 || A_23_P69242 || MAP:3p26.2 || SUMF1 -- sulfatase modi WID:6495539
WID:6475745 || A_23_P215275 || MAP: || -- CDNA clone IMAGE:658 WID:6475745
WID:6482576 || A_23_P94118 || MAP:8p12 || GTF2E2 -- general transc WID:6482576
WID:6493991 || A_24_P255645 || MAP:3q26.1 || IFT80 -- intraflagellar tra WID:6493991
WID:6479593 || A_23_P216071 || MAP: || -- Unknown || WID:6479593
WID:6498655 || A_23_P166336 || MAP:22q11.21 || TMEM191A -- transmer WID:6498655
WID:6587487 || A_23_P250813 || MAP:8p12 || WRN -- Werner syndrom WID:6587487
WID:6496038 || A_32_P127501 || MAP: || CACNB2 -- Calcium channe WID:6496038
WID:6479609 || A_24_P138713 || MAP:16q12.1 || HEATR3 -- HEAT repea WID:6479609
WID:6489325 || A_24_P924261 || MAP:6p24.1 || HERV-FRD -- HERV-FR WID:6489325
WID:6503608 || A_24_P34568 || MAP:2q21.1 || FAM123C -- family with s WID:6503608
WID:6511346 || A_23_P204998 || MAP:13q32.2 || FARP1 -- FERM, RhoGI WID:6511346
WID:6487400 || A_24_P269598 || MAP: || ENTPD4 -- Ectonucleoside WID:6487400
WID:6511651 || A_24_P922838 || MAP: || DDHD2 -- DDHD domain co WID:6511651
WID:6498633 || A_23_P136552 || MAP:4p16.3 || PIGG -- phosphatidylinos WID:6498633
WID:6508428 || A_32_P204722 || MAP:16q22.3 || LOC645726 -- PREDIC WID:6508428
WID:6513628 || A_24_P81900 || MAP:12p13.31 || SLC2A3 -- solute carrier WID:6513628
WID:6515773 || A_23_P15090 || MAP:16q12.1 || DNAJA2 -- DnaJ (Hsp40 WID:6515773
WID:6483335 || A_24_P15973 || MAP: || -- Unknown || WID:6483335
WID:6475004 || A_23_P124486 || MAP:15q24.2 || PTPN9 -- protein tyrosin WID:6475004
WID:6509838 || A_24_P270376 || MAP:13q14.12 || NUFIP1 -- nuclear fragi WID:6509838
WID:6518100 || A_23_P308073 || MAP:13q12.12 || SACS -- spastic ataxia WID:6518100
WID:6489731 || A_32_P222521 || MAP: || -- Transcribed locus || WID:6489731
WID:6475912 || A_23_P156826 || MAP:6p24.1 || C6orf105 -- chromosome WID:6475912
WID:6515479 || A_23_P67771 || MAP:2q35 || BARD1 -- BRCA1 associa WID:6515479
WID:6496211 || A_23_P95070 || MAP: || -- CDNA FLJ12345 fis, clon WID:6496211
WID:6491077 || A_23_P161769 || MAP:11q23.3 || FXYD2 -- FXYD domain WID:6491077
WID:6494785 || A_23_P162782 || MAP:13q33.3 || ARGLU1 -- arginine and WID:6494785
WID:6518257 || A_24_P289709 || MAP: || HBG2 -- Hemoglobin, gamn WID:6518257
WID:6512855 || A_23_P66473 || MAP:17q24.2 || PITPNC1 -- phosphatidy WID:6512855

WID:6510885 || A_32_P139505 || MAP:1p36.21 || KIAA1026 -- kazrin (KIA WID:6510885
WID:6501839 || A_23_P124585 || MAP: || ZNF93 -- Zinc finger protein WID:6501839
WID:6485801 || A_24_P208794 || MAP:3p21.31 || APEH -- N-acylaminoac WID:6485801
WID:6491302 || A_23_P38085 || MAP:16p13.2 || CARHSP1 -- calcium reg WID:6491302
WID:6485159 || A_24_P12539 || MAP:7p14.3 || KBTBD2 -- kelch repeat a WID:6485159
WID:6513420 || A_23_P162374 || MAP:12q24.13 || DDX54 -- DEAD (Asp-G WID:6513420
WID:6508315 || A_23_P51966 || MAP:1p33 || STIL -- SCL/TAL1 interrup WID:6508315
WID:6497290 || A_32_P204381 || MAP:16q13 || CIAPIN1 -- cytokine indu WID:6497290
WID:6478874 || A_23_P342910 || MAP: || KLHL15 -- Kelch-like 15 (Dr WID:6478874
WID:6500144 || A_24_P263259 || MAP:14q32.2 || CCDC85C -- coiled-coil WID:6500144
WID:6511591 || A_23_P193 || MAP:1p34.2 || DEM1 -- defects in morpho WID:6511591
WID:6505337 || A_23_P21882 || MAP: || -- Unknown || WID:6505337
WID:6502252 || A_23_P38365 || MAP:17q23.2 || TLK2 -- tousled-like kina WID:6502252
WID:6496619 || A_23_P30736 || MAP:6p21.32 || HLA-DOB -- major histoc WID:6496619
WID:6515484 || A_23_P354170 || MAP:3q29 || PIGX -- phosphatidylinosi WID:6515484
WID:6497231 || A_23_P40455 || MAP:21q22.13 || DSCR4 -- Down syndror WID:6497231
WID:6478172 || A_32_P71032 || MAP: || -- Transcribed locus || WID:6478172
WID:6495170 || A_23_P214658 || MAP:6p21.32 || PBX2 -- pre-B-cell leuke WID:6495170
WID:6484300 || A_32_P190036 || MAP: || LOC441964 -- Hypothetical WID:6484300
WID:6489873 || A_32_P185393 || MAP: || -- Transcribed locus || WID:6489873
WID:6486797 || A_23_P71926 || MAP:9q21.11 || PRKACG -- protein kinas WID:6486797
WID:6475710 || A_23_P106174 || MAP: || PSEN1 -- Presenilin 1 || WID:6475710
WID:6515702 || A_24_P69095 || MAP:5q13.3 || ENC1 -- ectodermal-neur WID:6515702
WID:6487542 || A_24_P384969 || MAP: || -- Homo sapiens, clone IMA WID:6487542
WID:6515798 || A_23_P68198 || MAP:2p25.3 || SH3YL1 -- SH3 domain c WID:6515798
WID:6585860 || A_23_P123234 || MAP: || -- Unknown || WID:6585860
WID:6488203 || A_24_P282043 || MAP:19q13.41 || ZNF28 -- zinc finger pro WID:6488203
WID:6489671 || A_32_P147969 || MAP: || -- MRNA; cDNA DKFZp586 WID:6489671
WID:6512059 || A_23_P136961 || MAP: || -- Unknown || WID:6512059
WID:6503560 || A_23_P71537 || MAP:8q13.2 || CSPP1 -- centrosome an WID:6503560
WID:6493619 || A_32_P130522 || MAP: || -- Transcribed locus || WID:6493619
WID:6512779 || A_23_P155009 || MAP:22q12.2 || RHBDD3 -- rhomboid do WID:6512779
WID:6499246 || A_23_P205370 || MAP:14q32.13 || ASB2 -- ankyrin repeat WID:6499246
WID:6508900 || A_23_P385851 || MAP:Xq26.2 || MBNL3 -- muscleblind-lil WID:6508900
WID:6504870 || A_23_P319557 || MAP: || C21orf89 -- Chromosome 2 WID:6504870
WID:6506717 || A_23_P54147 || MAP:14q22.1 || NIN -- ninein (GSK3B int WID:6506717
WID:6516739 || A_23_P27279 || MAP:18q21.31 || NEDD4L -- neural precu WID:6516739
WID:6501515 || A_24_P44931 || MAP:19p13.3 || MPND -- MPN domain co WID:6501515
WID:6496598 || A_24_P923483 || MAP: || SLC6A13 -- Solute carrier fa WID:6496598
WID:6504639 || A_24_P531312 || MAP: || -- Transcribed locus || WID:6504639
WID:6499709 || A_24_P370201 || MAP:19p13.3 || EBI3 -- Epstein-Barr viru WID:6499709
WID:6482994 || A_23_P125001 || MAP:17q25.3 || RAC3 -- ras-related C3 I WID:6482994
WID:6514921 || A_32_P97513 || MAP: || -- Unknown || WID:6514921
WID:6479668 || A_23_P149818 || MAP:10p14 || UPF2 -- UPF2 regulator (WID:6479668
WID:6479545 || A_24_P304760 || MAP:15q24.2 || SIN3A -- SIN3 homolog WID:6479545
WID:6507700 || A_32_P14721 || MAP:3p14.3 || DNAH12 -- dynein, axone WID:6507700
WID:6503931 || A_23_P157620 || MAP:8p21.3 || FAM160B2 -- family with WID:6503931
WID:6505646 || A_23_P129174 || MAP:15q23 || LRRC49 -- leucine rich re WID:6505646
WID:6493658 || A_23_P146325 || MAP:8q24.21 || DDEF1IT1 -- DDEF1 int WID:6493658
WID:6512511 || A_24_P728115 || MAP: || -- CDNA: FLJ21284 fis, clo WID:6512511
WID:6475412 || A_23_P106806 || MAP:16p13.3 || PRSS27 -- protease, se WID:6475412
WID:6503570 || A_23_P116578 || MAP:11q14.1 || PCF11 -- PCF11, cleava WID:6503570

WID:6481832	|| A_24_P404628	|| MAP:22q12.2	|| C22orf30 -- chromosom	WID:6481832
WID:6516216	|| A_23_P216556	|| MAP:9q31.3	|| EPB41L4B -- erythrocyte	WID:6516216
WID:6495244	|| A_24_P870463	|| MAP:	|| -- MRNA; cDNA DKFZp434	WID:6495244
WID:6513382	|| A_24_P556318	|| MAP:11p15.1	|| SAA3P -- serum amyloid	WID:6513382
WID:6476865	|| A_24_P70888	|| MAP:22q13.33	|| PLXNB2 -- plexin B2 (Pl	WID:6476865
WID:6488014	|| A_24_P919916	|| MAP:	|| PTF1A -- Pancreas specific	WID:6488014
WID:6492111	|| A_23_P384405	|| MAP:8p21.1	|| EXTL3 -- exostoses (mu	WID:6492111
WID:6502872	|| A_23_P109655	|| MAP:3q22.3	|| TMEM22 -- transmembra	WID:6502872
WID:6478969	|| A_23_P216806	|| MAP:9q21.11	|| APBA1 -- amyloid beta (	WID:6478969
WID:6505677	|| A_23_P3424	|| MAP:15q24.1	|| RPP25 -- ribonuclease P/	WID:6505677
WID:6477179	|| A_32_P47200	|| MAP:	|| DKFZp547E087 -- KIAA022(	WID:6477179
WID:6486734	|| A_23_P68511	|| MAP:20p13	|| ANGPT4 -- angiopoietin 4	WID:6486734
WID:6475251	|| A_24_P931598	|| MAP:	|| LMAN2 -- Lectin, mannose-	WID:6475251
WID:6514310	|| A_23_P39871	|| MAP:2q36.3	|| SLC19A3 -- solute carrier	WID:6514310
WID:6494405	|| A_24_P222165	|| MAP:	|| GAB1 -- GRB2-associated b	WID:6494405
WID:6479308	|| A_24_P759674	|| MAP:10q24.33	|| OBFC1 -- oligonucleotic	WID:6479308
WID:6487814	|| A_23_P163455	|| MAP:15q15.3	|| MAP1A -- microtubule-a	WID:6487814
WID:6484909	|| A_23_P254353	|| MAP:9q34.3	|| NOXA1 -- NADPH oxidas	WID:6484909
WID:6480580	|| A_32_P134772	|| MAP:2q31.2	|| LOC100130691 -- hypoth	WID:6480580
WID:6483615	|| A_24_P272761	|| MAP:9q33.2	|| DENND1A -- DENN/MAD	WID:6483615
WID:6482785	|| A_23_P112846	|| MAP:4q13.3	|| MTHFD2L -- methylenete	WID:6482785
WID:6586105	|| A_23_P123315	|| MAP:	|| -- CDNA clone IMAGE:482(	WID:6586105
WID:6514206	|| A_32_P193939	|| MAP:	|| -- CDNA FLJ41316 fis, clon	WID:6514206
WID:6491149	|| A_23_P305321	|| MAP:	|| C8orf58 -- Chromosome 8 c	WID:6491149
WID:6505437	|| A_24_P401921	|| MAP:	|| SLC24A1 -- Solute carrier fa	WID:6505437
WID:6477725	|| A_24_P795594	|| MAP:11q23.1	|| ALG9 -- asparagine-link	WID:6477725
WID:6513142	|| A_23_P115046	|| MAP:1p34.1	|| EIF2B3 -- eukaryotic tran	WID:6513142
WID:6486231	|| A_24_P925274	|| MAP:	|| HMG20A -- high-mobility gro	WID:6486231
WID:6514678	|| A_32_P202977	|| MAP:	|| -- Transcribed locus ||	WID:6514678
WID:6512999	|| A_23_P21457	|| MAP:3q26.1	|| NMD3 -- NMD3 homolog	WID:6512999
WID:6515953	|| A_24_P925124	|| MAP:	|| MBD1 -- Methyl-CpG bindin	WID:6515953
WID:6506763	|| A_24_P401686	|| MAP:17q21.33	|| CACNA1G -- calcium cl	WID:6506763
WID:6504873	|| A_24_P265346	|| MAP:17q21.2	|| KRT14 -- keratin 14 (KR	WID:6504873
WID:6491087	|| A_32_P123788	|| MAP:19q13.42	|| MBOAT7 -- membrane	WID:6491087
WID:6484171	|| A_32_P5150	|| MAP:	|| -- Unknown ||	WID:6484171
WID:6492439	|| A_32_P155416	|| MAP:1p34.1	|| ERI3 -- exoribonuclease	WID:6492439
WID:6500013	|| A_24_P221323	|| MAP:3q23	|| ZBTB38 -- zinc finger anc	WID:6500013
WID:6510110	|| A_23_P333420	|| MAP:22q13.2	|| RANGAP1 -- Ran GTPa	WID:6510110
WID:6488411	|| A_23_P54469	|| MAP:15q24.3	|| ISL2 -- ISL LIM homeobc	WID:6488411
WID:6509039	|| A_24_P44891	|| MAP:19p13.13	|| TNPO2 -- transportin 2 (	WID:6509039
WID:6479789	|| A_32_P33304	|| MAP:	|| ANK3 -- Ankyrin 3, node of F	WID:6479789
WID:6478384	|| A_24_P889103	|| MAP:	|| SUZ12P -- Suppressor of ze	WID:6478384
WID:6498031	|| A_23_P251002	|| MAP:	|| -- Unknown ||	WID:6498031
WID:6476759	|| A_24_P103744	|| MAP:	|| -- CDNA clone IMAGE:447	WID:6476759
WID:6513234	|| A_23_P77223	|| MAP:15q26.1	|| MESP1 -- mesoderm pos	WID:6513234
WID:6486363	|| A_24_P357709	|| MAP:3q27.2	|| VPS8 -- vacuolar protein	WID:6486363
WID:6518005	|| A_24_P326491	|| MAP:10p12.1	|| MKX -- mohawk homeol	WID:6518005
WID:6493784	|| A_32_P209148	|| MAP:12q22	|| LOC144486 -- hypothetic	WID:6493784
WID:6489062	|| A_23_P23947	|| MAP:10p11.23	|| MAP3K8 -- mitogen-acti	WID:6489062
WID:6474839	|| A_23_P167159	|| MAP:4q34.1	|| SCRG1 -- scrapie respor	WID:6474839
WID:6488657	|| A_23_P169017	|| MAP:	|| DEFB103A -- defensin, beta	WID:6488657
WID:6474860	|| A_24_P263310	|| MAP:15q26.3	|| FAM169B -- family with	WID:6474860

WID:6491412	|| A_32_P234591	|| MAP:	|| -- Transcribed locus ||	WID:6491412
WID:6491743	|| A_24_P391586	|| MAP:11q23.3	|| OAF -- OAF homolog (D	WID:6491743
WID:6479819	|| A_23_P18152	|| MAP:3p25.3	|| ATP2B2 -- ATPase, Ca+-	WID:6479819
WID:6498586	|| A_24_P162226	|| MAP:12q24.33	|| RIMBP2 -- RIMS bindin	WID:6498586
WID:6477776	|| A_23_P52109	|| MAP:	|| PRKAA2 -- Protein kinase, A	WID:6477776
WID:6502682	|| A_24_P78862	|| MAP:17p13.3	|| SLC43A2 -- solute carrie	WID:6502682
WID:6509948	|| A_32_P113736	|| MAP:	|| -- Transcribed locus ||	WID:6509948
WID:6488838	|| A_24_P204067	|| MAP:	|| CD58 -- CD58 molecule ||	WID:6488838
WID:6488410	|| A_23_P84860	|| MAP:3p14.2	|| FAM107A -- family with s	WID:6488410
WID:6517229	|| A_24_P929388	|| MAP:2q35	|| TMEM169 -- transmembr	WID:6517229
WID:6502639	|| A_32_P50587	|| MAP:	|| -- Transcribed locus ||	WID:6502639
WID:6514197	|| A_23_P127339	|| MAP:10q24.2	|| PI4K2A -- phosphatidylir	WID:6514197
WID:6503306	|| A_24_P50972	|| MAP:15q11.2	|| LOC283767 -- golgi auto	WID:6503306
WID:6491980	|| A_24_P623782	|| MAP:3p12.3	|| FLJ20518 -- PREDICTEI	WID:6491980
WID:6504174	|| A_32_P27479	|| MAP:19q13.42	|| NLRP11 -- NLR family,	WID:6504174
WID:6481577	|| A_24_P355693	|| MAP:11q13.5	|| ACER3 -- alkaline ceran	WID:6481577
WID:6487158	|| A_32_P101653	|| MAP:	|| -- Transcribed locus ||	WID:6487158
WID:6492757	|| A_23_P345778	|| MAP:11p14.1	|| C11orf46 -- chromosom	WID:6492757
WID:6511817	|| A_24_P698136	|| MAP:4p14	|| LOC344967 -- PREDICTI	WID:6511817
WID:6517384	|| A_23_P109393	|| MAP:22q11.21	|| ARVCF -- armadillo rep	WID:6517384
